# Dietary Fiber Ameliorates Lipopolysaccharide-Induced Intestinal Barrier Function Damage in Piglets by Modulation of Intestinal Microbiome

**DOI:** 10.1128/mSystems.01374-20

**Published:** 2021-04-06

**Authors:** Xiao Sun, Yalei Cui, Yingying Su, Zimin Gao, Xinying Diao, Ju Li, Xiaoyan Zhu, Defeng Li, Zhentian Li, Chengzhang Wang, Yinghua Shi

**Affiliations:** a College of Animal Science and Technology, Henan Agricultural University, Zhengzhou, China; b Henan Key Laboratory of Innovation and Utilization of Grassland Resources, Zhengzhou, China; c Henan Forage Engineering Technology Research Center, Zhengzhou, China; d Henan Yinfa Animal Husbandry Co., Xinzheng, China; University of California, San Diego

**Keywords:** dietary fiber, lipopolysaccharide, piglets, inflammatory reaction, intestinal barrier function, intestinal microflora

## Abstract

Weaning of piglets is accompanied by intestinal inflammation, impaired intestinal barrier function, and intestinal microflora disorder. Regulating intestinal microflora structure can directly or indirectly affect intestinal health and host growth and development. However, whether dietary fiber (DF) affects the inflammatory response and barrier function by affecting the intestinal microflora and its metabolites is unclear. In this study, we investigated the role of intestinal microflora in relieving immune stress and maintaining homeostasis using piglets with lipopolysaccharide (LPS)-induced intestinal injury as a model. DF improved intestinal morphology and barrier function, inhibited the expression of inflammatory signal pathways (Toll-like receptor 2 [TLR2], TLR4, and NF-κB) and proinflammatory cytokines (interleukin 1β [IL-1β], IL-6, and tumor necrosis factor alpha [TNF-α]), and upregulated the expression of barrier-related genes (encoding claudin-1, occludin, and ZO-1). The contents of proinflammatory cytokines (IL-1β, IL-6, and TNF-α) and the activity of diamine oxidase in plasma were decreased. Meanwhile, DF had a strong effect on the composition and function of intestinal microflora at different taxonomic levels, the relative abundances of cellulolytic bacteria and anti-inflammatory bacteria were increased, and the concentrations of propionate, butyrate, and total short-chain fatty acids (SCFAs) in intestinal contents were increased. In addition, the correlation analysis also revealed the potential relationship between metabolites and certain intestinal microflora, as well as the relationship between metabolites and intestinal morphology, intestinal gene expression, and plasma cytokine levels. These results indicate that DF improves intestinal barrier function, in part, by altering intestinal microbiota composition and increasing the synthesis of SCFAs, which subsequently alleviate local and systemic inflammation.

**IMPORTANCE** Adding DF to the diet of LPS-challenged piglets alleviated intestinal and systemic inflammation, improved intestinal barrier function, and ultimately alleviated the growth retardation of piglets. In addition, the addition of DF significantly increased the relative abundance of SCFA-producing bacteria and the production of SCFAs. We believe that the improvement of growth performance of piglets with LPS-induced injury can be attributed to the beneficial effects of DF on intestinal microflora and SCFAs, which reduced the inflammatory response in piglets, improving intestinal barrier function and enhancing body health. These research results provide a theoretical basis and guidance for the use of specific fiber sources in the diet to improve intestinal health and growth performance of piglets and thus alleviate weaning stress. Our data also provide insights for studying the role of DF in regulating gastrointestinal function in human infants.

## INTRODUCTION

During weaning, piglets are susceptible to infection by various pathogens and nonpathogens in the environment due to the immature development of the digestive system and immune system, the pressure caused by mother-to-child separation, and the sudden transformation from easily digestible milk to less digestible solid feed, leading to stress and resulting in reduced production performance and increased diarrhea rate, affecting the normal growth of piglets (1–3). Under immune stress, excessive secretion of inflammatory cytokines in the immune system, such as interleukin-1β (IL-1β), interleukin-6 (IL-6), and tumor necrosis factor-α (TNF-α), leads to damage of intestinal structure, digestion, absorption, and barrier function, thus increasing the risk of intestinal diseases such as intestinal inflammation and diarrhea (4–8). Previous studies showed that lipopolysaccharide (LPS) stimulation could cause immune cells to release a large number of inflammatory cytokines, and the increase of inflammatory cytokines could activate the hypothalamus-pituitary-adrenal axis and inhibit the growth axis, resulting in growth inhibition (9). Therefore, the immune stress model induced by LPS can be established to simulate weaning stress of piglets. Relying on nutritional regulation to improve the intestinal microflora and then regulate the secretion of inflammatory cytokines is of great significance for alleviating immune stress (10). At present, antibiotics play an important role in preventing intestinal function damage caused by early weaning stress (2), but due to the problems of drug resistance in bacteria and residual antibiotics in food (11), it is of great significance for the animal husbandry and food safety industries to find substitutes for antibiotics to maintain the health of piglets during the weaning period and protect public health (12). It has been found that dietary fiber (DF) is an effective substitute for growth promoters (13, 14), as it can regulate intestinal microflora, improve intestinal health, and thereby improve growth performance and reduce postweaning diarrhea (15, 16).

Intestinal microorganisms have extensive biological effects on the growth and health of humans and animals (17, 18). The interaction between members of the intestinal microflora and the interaction between intestinal microorganisms and hosts can regulate the biological processes that are vital to the health of the host, including metabolism of nutrients and energy in diet (19, 20), intestinal barrier function (21), maturation of the immune system (22–24), cell proliferation and differentiation (25, 26), and growth performance (27). The composition of intestinal microflora can be affected by stress, diet, age, lifestyle, and various environmental factors, which in turn can directly or indirectly affect the host’s nutritional metabolism, immune response, and intestinal homeostasis (28–30), shaping the interaction between intestinal microflora and host (31). DF can be degraded by intestinal microbes to produce short-chain fatty acids (SCFAs), thereby improving immune function and intestinal health (32–34). Therefore, it is necessary to add appropriate amounts of DF to the diet to regulate the host’s intestinal health. However, different dietary fiber sources may play different roles in regulating bacterial communities (35, 36). At present, there are few studies on the sources of DF added to the diet of weaned piglets. At the same time, some studies have shown that the early succession of intestinal microflora has a permanent impact on the metabolism of the host (37), and its early colonization of the intestine determines the host’s immune ability in the later stages of life (38). Therefore, it may be an effective strategy to regulate the development of early intestinal microflora through DF to maintain the long-term health of the host.

We hypothesized that DF could improve intestinal health by changing intestinal bacterial communities and metabolites. Due to the similarities in the digestion and metabolism of nutrients, intestinal anatomy, physiology, and microbial ecosystem between humans and pigs (39, 40), pigs are also often used as animal models to assess the interaction between gut microflora and host health, and they can exhibit diseases similar to those of humans, such as necrotizing enterocolitis (NEC) and weaning diarrhea (39). Therefore, using pigs as an animal model to study the pathophysiology of the human gastrointestinal tract is also of great significance to evaluate the effects of dietary fiber on the host’s intestinal microbes and metabolites (41). Previous studies reported that DF was mainly fermented in the hindgut of humans and other monogastric animals (16, 42). However, recent studies have shown that a large number of fiber components are also degraded in the foregut of monogastric animals (43–45). At the same time, since the ileum is the site of the interaction among mucosal cells, microflora, and nutrients (46), there is little information about how DF affects the bacterial community and SCFA production in the foregut. Therefore, in this experiment, piglets were injected with LPS to simulate immune stress (47) to study whether DF could alleviate LPS-induced immune stress by improving intestinal health by regulating intestinal microflora and SCFAs.

## RESULTS

### Growth performance and diarrheal conditions.

There was no significant difference in average daily feed intake (ADFI), average daily gain (ADG), and feed-to-gain ratio (F:G) between the groups from day 1 to 15 (*P > *0.05). From day 16 to 22, LPS stimulation significantly decreased the ADG of piglets (*P < *0.05), and the F:G tended to increase (*P = *0.053); after addition of alfalfa fiber or commercial fiber, the F:G of piglets decreased (*P > *0.05). From the 23rd to 28th day, the ADFI of piglets in the group that received LPS showed a tendency to decrease compared with that in the basal diet control (CK) group (*P = *0.061). The addition of alfalfa fiber significantly increased the ADFI and ADG of piglets (*P < *0.05), and the F:G tended to decrease (*P = *0.073); after addition of commercial fiber, compared with LPS group, the ADG of piglets increased significantly (*P < *0.05), and the F:G tended to decrease (*P = *0.093). After addition of commercial fiber, compared with the group receiving LPS and alfalfa fiber (LPS+AF group), the ADFI of piglets decreased significantly (*P < *0.05), and the ADG tended to decrease (*P = *0.054). During the whole trial period (from day 1 to 28), LPS stimulation significantly decreased the ADG and increased the F:G of piglets (*P < *0.05); the addition of alfalfa fiber significantly increased the ADG (*P < *0.05), while the ADFI tended to increase (*P = *0.100), and the F:G decreased significantly (*P < *0.05). the addition of commercial fiber significantly increased the ADG of piglets and decreased the F:G (*P < *0.05) (Table 1).

**TABLE 1:** Growth performance of piglets in different groups^*a*^

Parameter and period (days)	Value for piglet group			
	CK	LPS	LPS+AF	LPS+CF
ADFI (g)
1 to 15	532.92 ± 57.94	499.58 ± 12.17	522.17 ± 55.25	539.25 ± 28.83
16 to 22	760.36 ± 148.45	689.38 ± 122.32	720.27 ± 16.88	650.27 ± 13.12
23 to 28	1,037.81 ± 109.27 ab	931.02 ± 49.03 b	1,131.42 ± 7.53 a	969.17 ± 4.58 b
1 to 28	674.90 ± 108.94	622.13 ± 34.28	719.16 ± 18.52	663.16 ± 54.17

ADG (g)
1 to 15	315.33 ± 24.45	303.06 ± 88.94	307.34 ± 50.13	323.78 ± 48.70
16 to 22	452.38 ± 89.43 a	295.71 ± 103.10 b	312.71 ± 0.21 b	328.37 ± 80.76 b
23 to 28	468.75 ± 100.45 bc	402.78 ± 13.89 c	640.63 ± 15.63 a	538.19 ± 42.10 ab
1 to 28	382.47 ± 51.41 a	295.55 ± 14.90 b	395.46 ± 0.37 a	370.87 ± 53.80 a

F:G
1 to 15	1.69 ± 0.06	1.74 ± 0.46	1.71 ± 0.18	1.69 ± 0.21
16 to 22	1.68 ± 0.01	2.46 ± 0.59	2.30 ± 0.05	2.08 ± 0.60
23 to 28	2.30 ± 0.61	2.32 ± 0.19	1.77 ± 0.03	1.81 ± 0.13
1 to 28	1.76 ± 0.07 b	2.11 ± 0.01 a	1.82 ± 0.05 b	1.80 ± 0.13 b

a Values are means ± SD. Values with different letters in a row are significantly different at a *P* value of <0.05. CK, basal diet (control); LPS, basal diet plus LPS; LPS+AF, basal diet plus LPS plus alfalfa fiber; LPS+CF, basal diet plus LPS plus commercial fiber.

LPS stimulation significantly increased the diarrhea rate and diarrhea index of piglets (*P < *0.05); the addition of alfalfa fiber or commercial fiber significantly reduced the diarrhea rate and diarrhea index of piglets (*P < *0.05) (Table 2).

**TABLE 2:** Diarrhea of piglets in different groups^*a*^

Piglet group	Diarrhea rate (%)	Diarrhea index
CK	3.57 ± 1.79 b	0.75 ± 0.33 b
LPS	6.55 ± 1.03 a	1.54 ± 0.26 a
LPS+AF	3.57 ± 0.00 b	0.88 ± 0.25 b
LPS+CF	2.98 ± 1.03 b	0.63 ± 0.33 b

a Values are means ± SD. Values with different letters in a column are significantly different at a *P* value of <0.05. CK, basal diet (control); LPS, basal diet plus LPS; LPS+AF, basal diet plus LPS plus alfalfa fiber; LPS+CF, basal diet plus LPS plus commercial fiber.

### Intestinal morphology.

**FIG 1:**
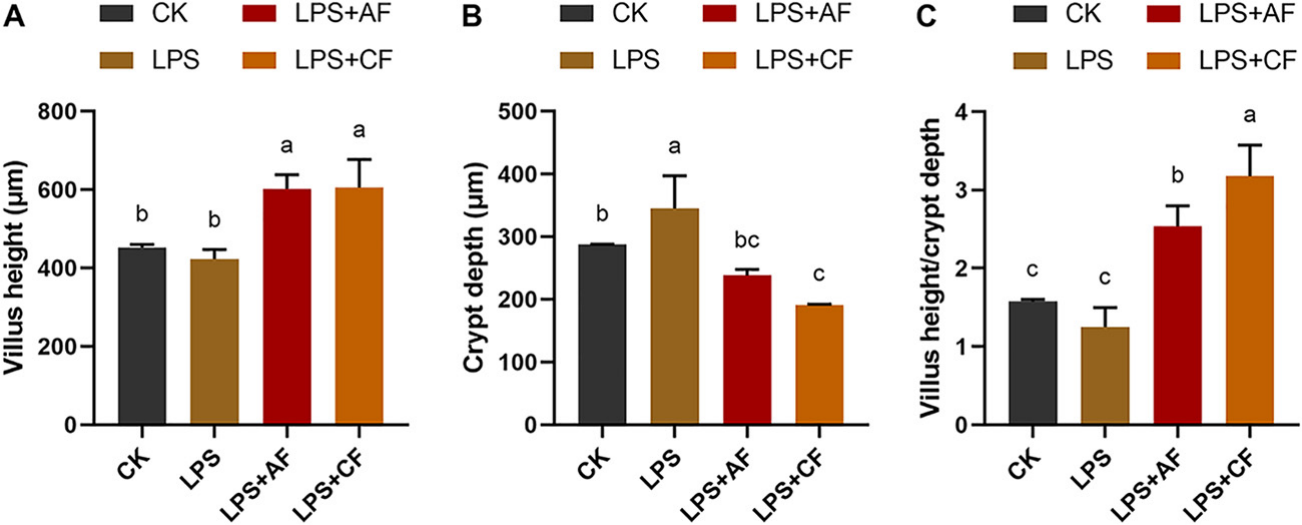
Changes in intestinal morphology of piglets in different groups. (A) Villus height. (B) Crypt depth. (C) Villus height/crypt depth. The data are means and SD. Means with different letters are significantly different (*P < *0.05). CK, basal diet (control); LPS, basal diet plus LPS; LPS+AF, basal diet plus LPS plus alfalfa fiber; LPS+CF, basal diet plus LPS plus commercial fiber.

LPS stimulation significantly increased the crypt depth (CD) of the ileum of piglets (*P < *0.05); after addition of alfalfa fiber or commercial fiber, the villus height (VH) and VH-CD ratio (VH:CD) in the LPS+AF group and the group receiving LPS plus commercial fiber (LPS+CF group) were significantly higher than those in the LPS group, while the CD was significantly decreased (*P < *0.05). After addition of commercial fiber, compared with LPS+AF group, the VH of ileum in the LPS+CF group showed no significant difference (*P > *0.05), but the CD tended to decrease (*P = *0.059), and the VH:CD increased significantly (*P < *0.05) (Fig. 1).

### Expression of intestinal barrier- and inflammation-related genes.

**FIG 2:**
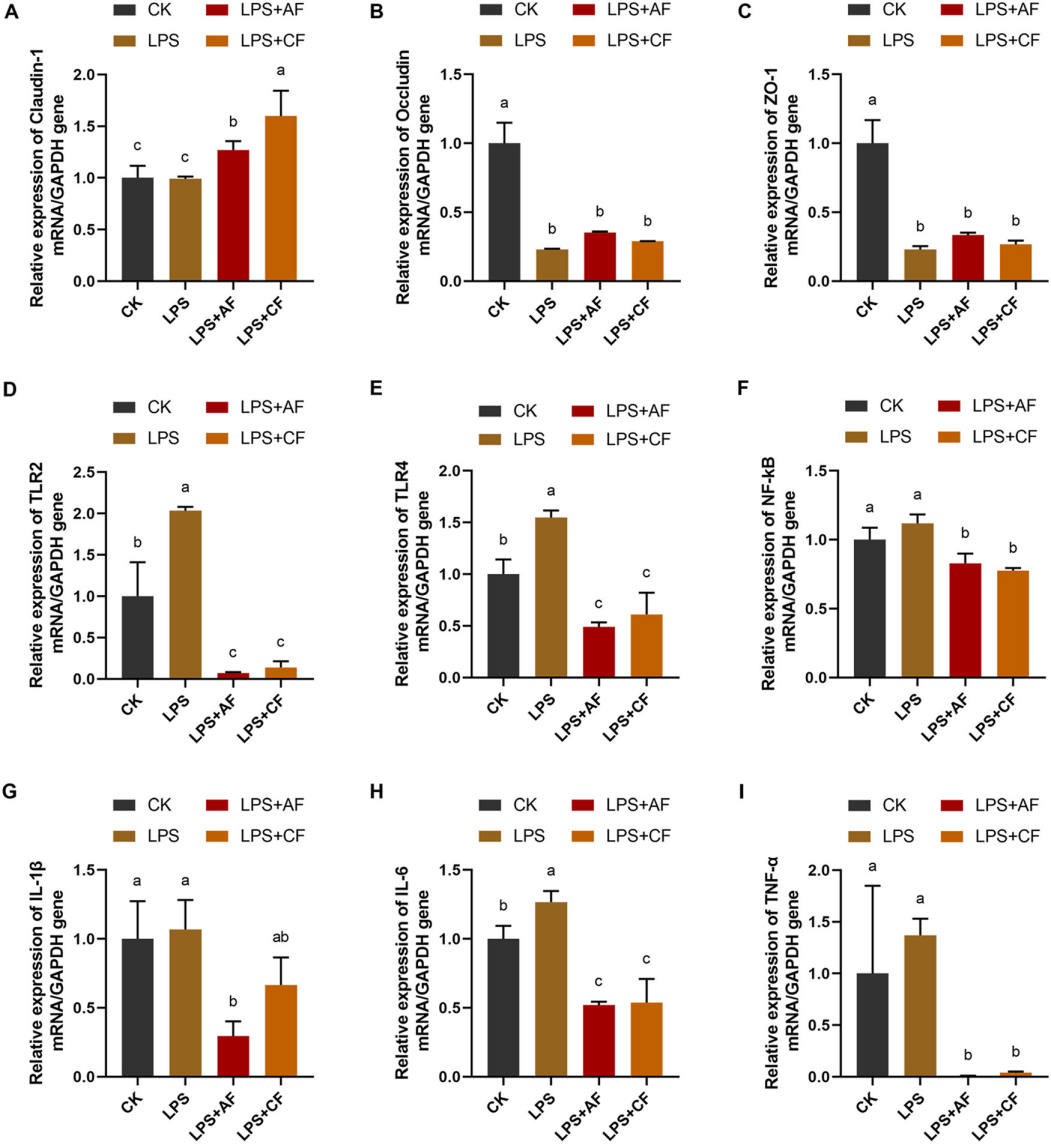
mRNA expression levels of intestinal barrier, immune, and inflammatory cytokines of piglets in different groups. The fold change in mRNA expression relative to GAPDH for claudin-1 (A) occludin (B), ZO-1 (C), TLR2 (D), TLR4 (E), NF-κB (F), IL-1β (G), IL-6 (H), and TNF-α (I) is shown. The data are means and SD. Means with different letters are significantly different (*P < *0.05). CK, basal diet (control); LPS, basal diet plus LPS; LPS+AF, basal diet plus LPS plus alfalfa fiber; LPS+CF, basal diet plus LPS plus commercial fiber.

LPS stimulation significantly decreased the expression of occludin and ZO-1 in the ileum of piglets (*P < *0.05). The addition of alfalfa fiber significantly increased the expression of claudin-1 (*P < *0.05), and the expression of occludin tended to increase (*P = *0.079); the expression of claudin-1 was significantly increased by addition of commercial fiber (*P < *0.05), while the expression of occludin and ZO-1 was increased (*P > *0.05). After addition of commercial fiber, compared with the LPS+AF group, the expression of claudin-1 in the LPS+CF group was significantly increased (*P < *0.05), while the expression of occludin and ZO-1 was not significantly different (*P > *0.05) (Fig. 2A to C).

LPS stimulation significantly increased the expression of Toll-like receptor 2 (TLR2), TLR4, and IL-6 in the ileum of piglets (*P < *0.05), and the expression of nuclear factor-kB (NF-κB) tended to increase (*P = *0.059). The addition of alfalfa fiber significantly decreased the expression of TLR2, TLR4, NF-κB, IL-1β, IL-6, and TNF-α (*P < *0.05); the addition of commercial fiber significantly decreased the expression of TLR2, TLR4, NF-κB, IL-6, and TNF-α (*P < *0.05), and the expression of IL-1β tended to decrease (*P = *0.061). After addition of commercial fiber, there was no significant difference in the expression of ileal inflammation-related genes in the LPS+CF group compared with the LPS+AF group (*P > *0.05) (Fig. 2D to I).

### Concentration of plasma barrier, immune, and inflammatory factors.

**FIG 3:**
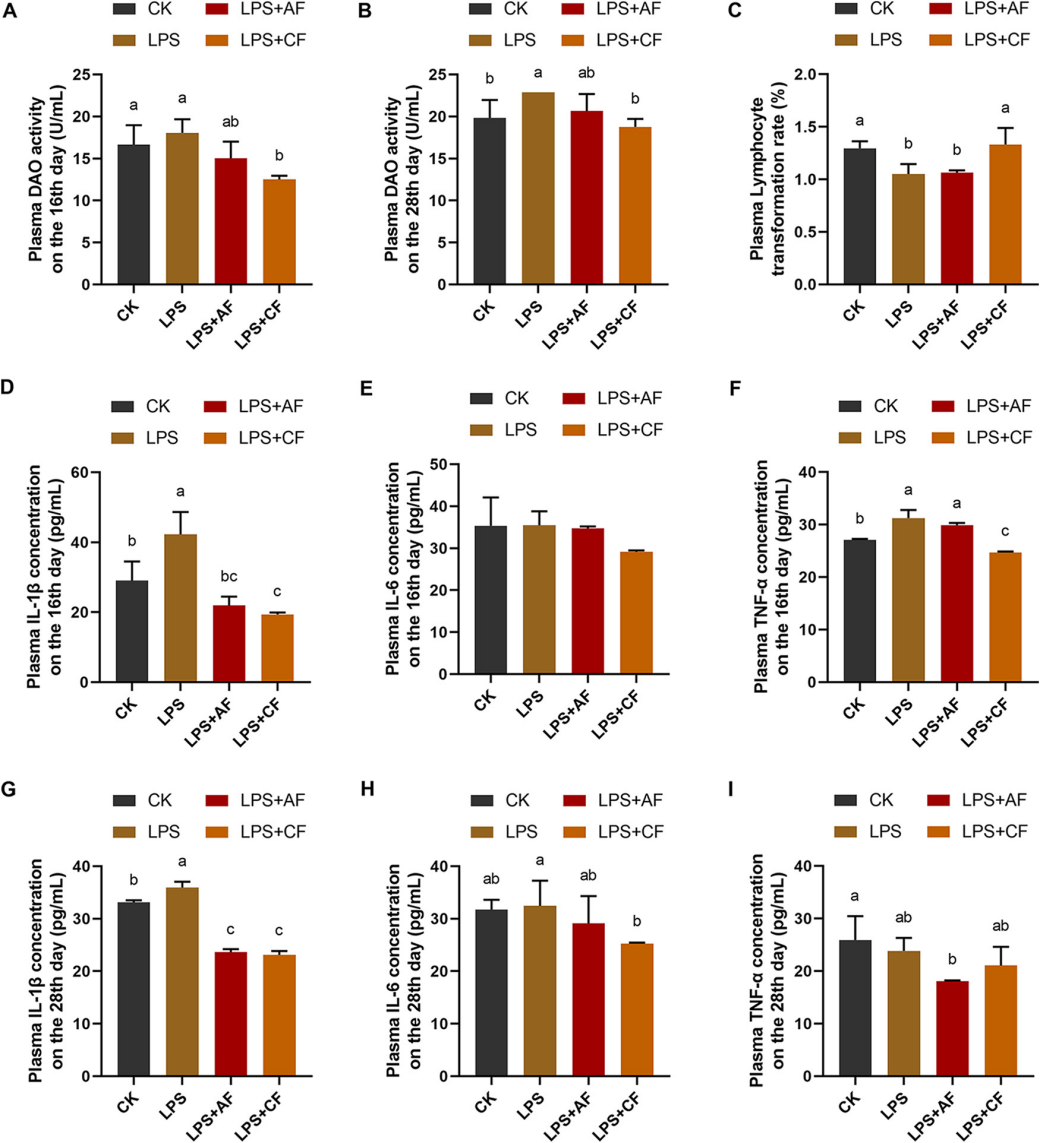
Plasma barrier, immune, and inflammatory cytokine concentrations of piglets in different groups. (A) DAO activity on the 16th day. (B) DAO activity on the 28th day. (C) Lymphocyte transformation rate. (D) IL-1β concentration on the 16th day. (E) IL-6 concentration on the 16th day. (F) TNF-α concentration on the 16th day. (G) IL-1β concentration on the 28th day. (H) IL-6 concentration on the 28th day. (I) TNF-α concentration on the 28th day. The data are means and SD. Means with different letters are significantly different (*P < *0.05). CK, basal diet (control); LPS, basal diet plus LPS; LPS+AF, basal diet plus LPS plus alfalfa fiber; LPS+CF, basal diet plus LPS plus commercial fiber.

LPS stimulation significantly increased plasma diamine oxidase (DAO) activity in piglets on day 28 (*P < *0.05); after addition of alfalfa fiber, the plasma DAO activity of LPS+AF group tended to decrease on day 16 (*P = *0.066). The addition of commercial fiber significantly decreased plasma DAO activity on day 16 and day 28 (*P < *0.05) (Fig. 3A and B).

LPS stimulation significantly decreased the lymphocyte transformation rate of peripheral blood in piglets (*P < *0.05). After addition of alfalfa fiber, the lymphocyte transformation rate of peripheral blood in the LPS+AF group increased (*P > *0.05); the addition of commercial fiber significantly increased the lymphocyte transformation rate of peripheral blood (*P < *0.05). After addition of commercial fiber, the lymphocyte transformation rate of peripheral blood in the LPS+CF group was significantly higher than that in the LPS+AF group (*P < *0.05) (Fig. 3C).

LPS stimulation significantly increased plasma IL-1β concentration on day 16, plasma TNF-α concentration on day 16, and plasma IL-1β concentration on day 28 in piglets (*P < *0.05). While the addition of alfalfa fiber significantly decreased the plasma IL-1β concentration on day 16 and day 28 (*P < *0.05), the plasma TNF-α concentration on day 16 and day 28 tended to decrease (*P = *0.078, *P = *0.055). The addition of commercial fiber significantly decreased the plasma IL-1β concentration on day 16 and day 28, plasma TNF-α concentration on day 16, and plasma IL-6 concentration on day 28 (*P < *0.05), and the plasma IL-6 concentration on day 16 showed a decreasing trend (*P = *0.073). After the addition of commercial fiber, compared with the LPS+AF group, the plasma TNF-α concentration of the LPS+CF group on day 16 was significantly decreased (*P < *0.05) (Fig. 3D to I).

### SCFA concentration in intestinal contents.

After LPS stimulation, compared with the CK group, the concentration of acetate in ileal contents of piglets tended to decrease (*P = *0.092), the concentration of propionate was decreased (*P > *0.05), and the concentrations of isobutyrate, butyrate, isovalerate, valerate, straight-chain fatty acids (StCFAs), branched-chain fatty acids (BCFAs), and total short-chain fatty acids (ToSCFAs) were significantly decreased (*P < *0.05). After addition of alfalfa fiber, the concentrations of acetate, propionate, isobutyrate, butyrate, isovalerate, valerate, StCFAs, BCFAs, and ToSCFAs in ileal contents were significantly higher than those in the LPS group (*P < *0.05). After addition of commercial fiber, compared with the LPS group, the concentrations of various short-chain fatty acids were increased (*P > *0.05). After addition of commercial fiber, compared with the LPS+AF group, the levels of acetate, propionate, butyrate, StCFAs, and ToSCFAs in ileal contents were decreased significantly (*P < *0.05), and the concentration of isobutyrate tended to decrease (*P = *0.087), while there was no significant difference in the concentrations of valerate, isovalerate, and BCFAs (*P > *0.05) (Table 3).

**TABLE 3:** Concentrations of SCFAs in the intestinal contents of piglets in different groups

SCFA	Concn (μg/g) in piglet group^*a*^			
	CK	LPS	LPS+AF	LPS+CF
Acetate	99.35 ± 12.80 ab	62.91 ± 3.61 b	116.38 ± 42.94 a	63.10 ± 12.14 b
Propionate	5.03 ± 1.50 b	3.65 ± 0.55 bc	7.08 ± 1.32 a	2.52 ± 0.24 c
Butyrate	34.14 ± 16.06 a	8.09 ± 2.01 b	26.41 ± 2.25 a	9.06 ± 3.83 b
Valerate	184.87 ± 3.21 a	31.17 ± 6.35 c	57.99 ± 14.51 b	38.75 ± 19.97 bc
Isobutyrate	71.67 ± 5.00 a	21.40 ± 0.83 c	33.88 ± 9.42 b	25.27 ± 1.65 bc
Isovalerate	49.37 ± 24.39 a	14.67 ± 4.38 b	41.42 ± 8.75 a	26.80 ± 5.35 ab
StCFAs	323.39 ± 15.78 a	105.82 ± 6.60 c	207.85 ± 43.57 b	113.43 ± 14.31c
BCFAs	121.04 ± 27.60 a	36.06 ± 5.21 c	75.30 ± 18.06 b	52.07 ± 4.30 bc
ToSCFAs	444.43 ± 15.24 a	141.88 ± 10.36 c	283.15 ± 27.52 b	165.50 ± 12.21 c

a Values are means ± SD. Values with different letters in a row are significantly different at a *P* value of <0.05. CK, basal diet (control); LPS, basal diet plus LPS; LPS+AF, basal diet plus LPS plus alfalfa fiber; LPS+CF, basal diet plus LPS plus commercial fiber.

### Overall structure of the intestinal microflora.

The microbiota composition in ileal mucosa of piglets was analyzed by the Illumina HiSeq sequencing system. After size filtering, quality control, and chimera checking, a total of 631,800 high-quality 16S rRNA gene sequences were amplified from 12 ileal mucosa samples, with an average of 52,650 high-quality sequences per sample; 54,409 ± 3,182, 55,763 ± 11,274, 50,874 ± 6,035, and 49,554 ± 3,763 sequences were observed in the CK, LPS, LPS+AF, and LPS+CF groups, respectively. A total of 5,627 operational taxonomic units (OTUs) were obtained at a sequence similarity level of 97% and were divided into 50, 131, 268, 518, 1,191, and 2,354 known groups at the phylum, class, order, family, genus, and species levels, respectively. A total of 895 OTUs shared by the four groups were identified, accounting for 15.9% of all sequences. Different numbers of unique OTUs, 573, 310, 772, and 1,041 for the CK, LPS, LPS+AF, and LPS+CF groups, respectively, were observed. In addition, there were 1,101, 1,334, 1,371, and 2,237 OTUs shared by the CK and LPS groups, LPS and LPS+AF groups, LPS and LPS+CF groups, and LPS+AF and LPS+CF groups, respectively (Fig. S1A).

10.1128/mSystems.01374-20.3 FIG S1 Overall structure of intestinal microflora of piglets in different groups. (A) Venn diagrams at the OTU level. (B) Rarefaction curves based on the Sobs index. (C) Rarefaction curves based on the Chao index. (D) Alpha diversity determined by the Sobs index. (E) Alpha diversity determined by the Chao index. (F) Alpha diversity determined by the Shannon index. The data are means ± SD. *, *P < *0.05. CK, basal diet (control); LPS, basal diet plus LPS; LPS+AF, basal diet plus LPS plus alfalfa fiber; LPS+CF, basal diet plus LPS plus commercial fiber. Download FIG S1, TIF file, 0.8 MB. Copyright © 2021 Sun et al. 2021 Sun et al. https://creativecommons.org/licenses/by/4.0/ This content is distributed under the terms of the Creative Commons Attribution 4.0 International license.

Using the abundances of bacterial OTUs across samples, we explored the global effects of DF on intestinal bacterial diversity. As shown, the quantity of observed species increased as the sequencing depth increased. The ends of the rarefaction curves tapered off with increasing numbers of sequencing per sample, indicating an adequate sequencing depth to investigate the dominant bacterial populations. Statistical analysis on the alpha diversity at the OTU level was conducted. The results showed that the Sobs (observed richness) index, Chao index, and Shannon index of the LPS group were lower than those of the CK group, but there was no statistical significance (*P > *0.05). After addition of alfalfa fiber, compared with the LPS group, the Sobs index and Chao index tended to increase (*P = *0.080 and *P = *0.100); after addition of commercial fiber, the Sobs index, Chao index, and Shannon index were increased significantly compared with the LPS group (*P < *0.05). after addition of commercial fiber, compared with the LPS+AF group, Sobs index, Chao index, and Shannon index were increased, but there was no statistical significance (*P > *0.05) (Fig. S1B to F).

### Composition and difference of intestinal microflora.

The relative abundance of microflora at the phylum level and genus level is shown in Fig. S2A and B. At the phylum level, 8 of the 50 phyla accounted for more than 1% of the total sequences (*Firmicutes*, *Proteobacteria*, *Tenericutes*, *Bacteroidetes*, *Actinobacteria*, *Chloroflexi*, *Cyanobacteria*, *Acidobacteria*), and *Firmicutes*, *Proteobacteria*, *Tenericutes*, and *Bacteroidetes* were most dominant, accounting for 30.74%, 22.57%, 18.40%, and 12.95% of the total sequences, respectively. At the genus level, the abundance of 17 genera accounted for more than 1% of the total sequences, and the dominant genera were *Lactobacillus* (phylum *Firmicutes*), *Mycoplasma* (phylum *Tenericutes*), and a genus annotated as “*norank_f__Bacteroidales_S24-7_group*” of the phylum *Bacteroidetes*, accounting for 6.05%, 18.27%, and 5.80% of the total sequence, respectively.

10.1128/mSystems.01374-20.4 FIG S2 Composition and difference of intestinal microflora of piglets in different groups. (A) Microbial composition at the phylum level. (B) Microbial composition at the genus level. (C) LEfSe analysis between CK and LPS groups. (D) LEfSe analysis between LPS and LPS+AF groups. (E) LEfSe analysis between LPS and LPS+CF groups. (F) Linear discriminant analysis (LDA) score between CK and LPS groups. (G) LDA score between LPS and LPS+AF groups. (H) LDA score between LPS and LPS+CF groups. CK, basal diet (control); LPS, basal diet plus LPS; LPS+AF, basal diet plus LPS plus alfalfa fiber; LPS+CF, basal diet plus LPS plus commercial fiber. Download FIG S2, TIF file, 2.2 MB. Copyright © 2021 Sun et al. 2021 Sun et al. https://creativecommons.org/licenses/by/4.0/ This content is distributed under the terms of the Creative Commons Attribution 4.0 International license.

**FIG 4:**
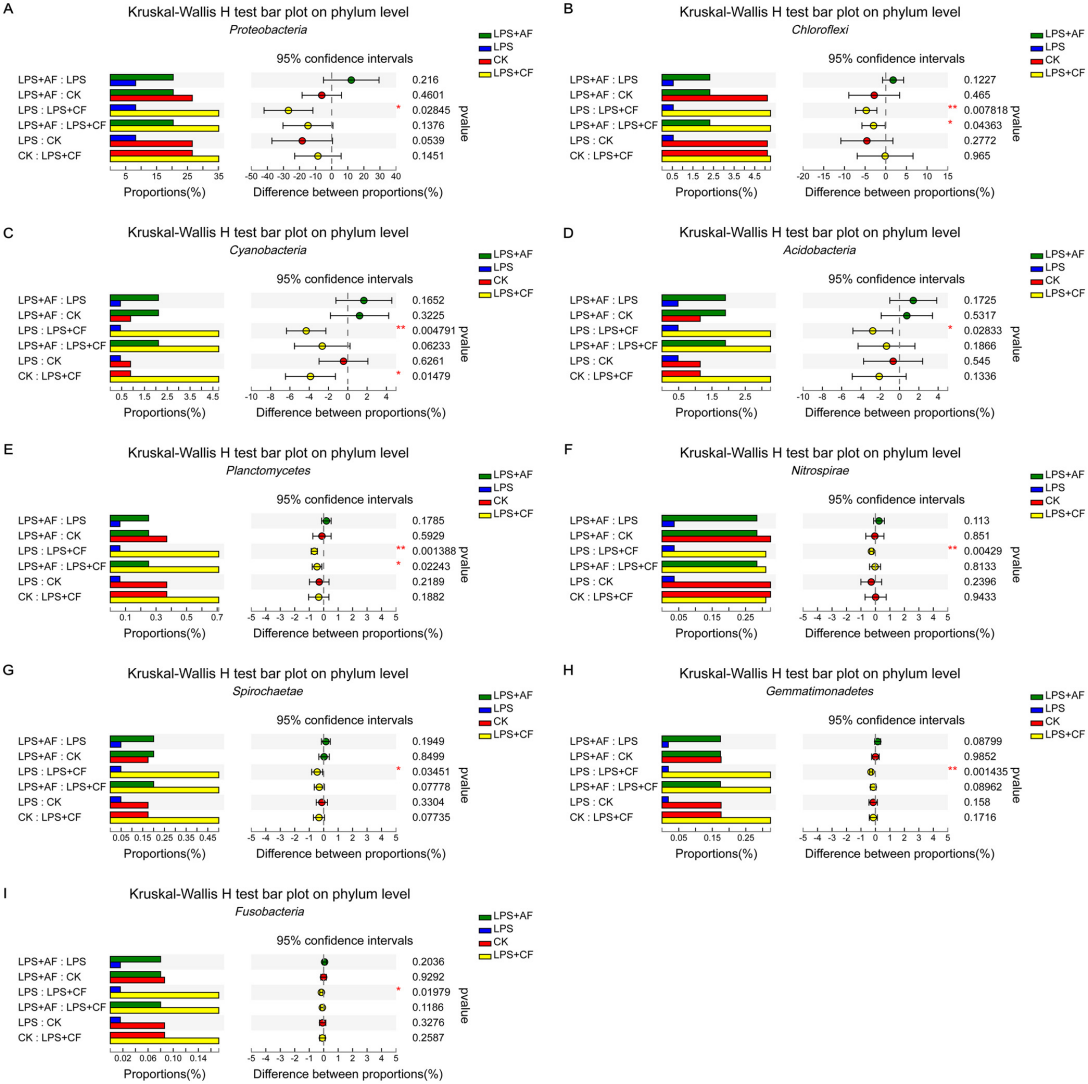
Changes in the intestinal microflora of piglets in different groups at the phylum level. (A) *Proteobacteria*. (B) *Chloroflexi*. (C) *Cyanobacteria*. (D) *Acidobacteria*. (E) *Planctomycetes*. (F) *Nitrospirae*. (G) *Spirochaeta*. (H) *Gemmatimonadetes*. (I) *Fusobacteria*. The data are means ± SD. *, *P < *0.05; **, *P < *0.01. CK, basal diet (control); LPS, basal diet plus LPS; LPS+AF, basal diet plus LPS plus alfalfa fiber; LPS+CF, basal diet plus LPS plus commercial fiber.

At the phylum level, after LPS stimulation, compared with the CK group, the relative abundance of *Proteobacteria* tended to decrease (*P = *0.054). After addition of alfalfa fiber, compared with the LPS group, the relative abundance of *Gemmatimonadetes* tended to increase (*P = *0.088); after addition of commercial fiber, compared with the LPS group, *Proteobacteria*, *Chloroflexi*, *Cyanobacteria*, *Acidobacteria*, *Planctomycetes*, *Nitrospirae*, *Spirochaeta*, *Gemmatimonadetes*, and *Fusobacteria* were significantly increased (*P < *0.05). After addition of commercial fiber, compared with the LPS+AF group, the relative abundance of *Chloroflexi* was increased significantly (*P < *0.05) and the relative abundance of *Gemmatimonadetes* tended to increase (*P = *0.090) (Fig. 4).

**FIG 5:**
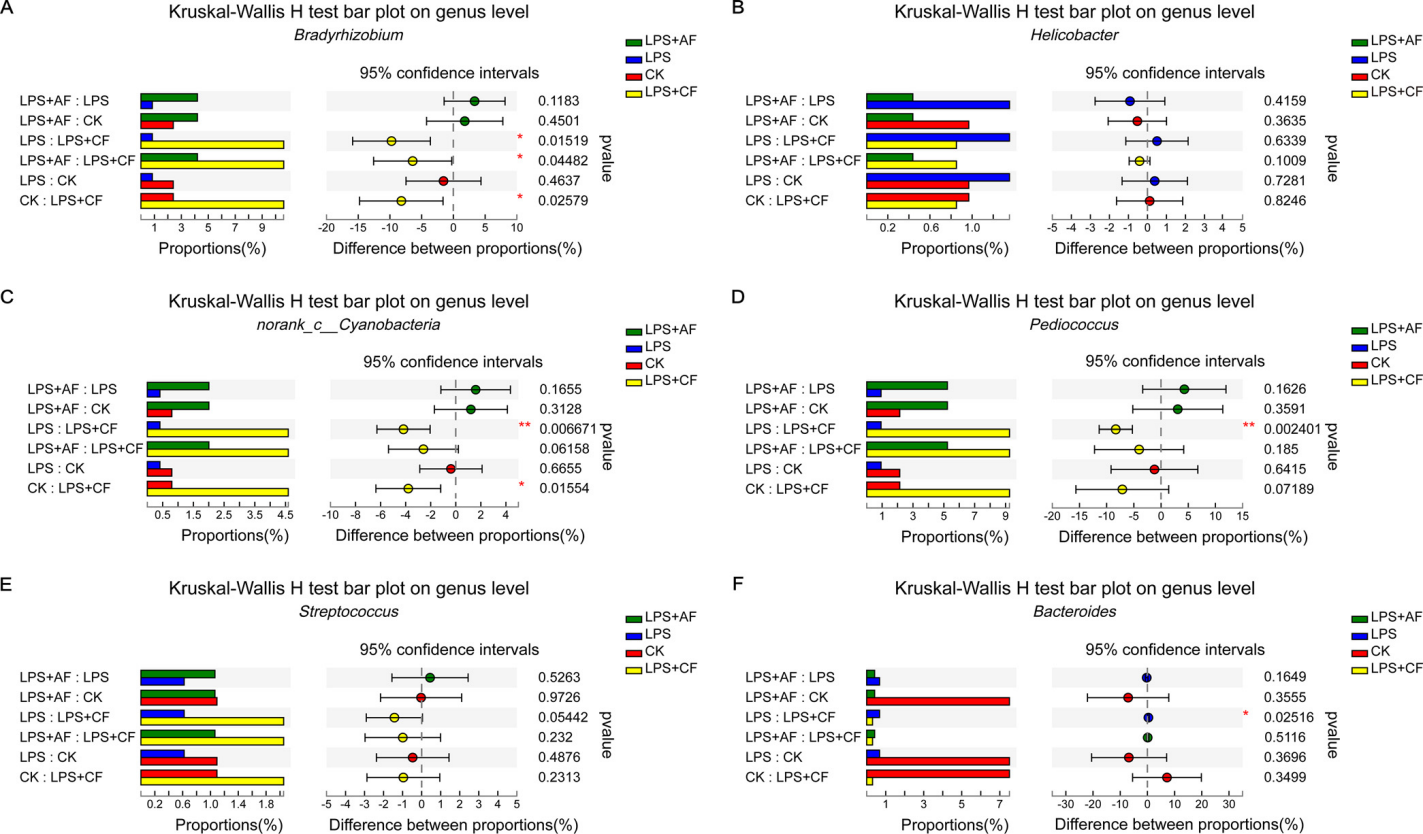
Changes in the intestinal microflora of piglets in different groups at the genus level. (A) *Bradyrhizobium*. (B) *Helicobacter*. (C) *norank_c__Cyanobacteria*. (D) *Pediococcus*. (E) *Streptococcus*. (F) *Bacteroide*. The data are means ± SD. *, *P < *0.05; **, *P < *0.01. CK, basal diet (control); LPS, basal diet plus LPS; LPS+AF, basal diet plus LPS plus alfalfa fiber; LPS+CF, basal diet plus LPS plus commercial fiber.

At the genus level, after LPS stimulation, the relative abundance of *Bradyrhizobium* was decreased compared with that in the CK group (*P > *0.05). After addition of alfalfa fiber, compared with the LPS group, the relative abundances of *Bradyrhizobium* and *Pediococcus* were increased (*P > *0.05), and the relative abundance of *Bacteroides* was decreased (*P > *0.05). After addition of commercial fiber, compared with the LPS group, the relative abundances of *Bradyrhizobium*, *Pediococcus*, and sequences annotated as *norank_c__Cyanobacteria* were increased significantly (*P < *0.05), the relative abundance of *Streptococcus* tended to increase (*P = *0.054), and the relative abundance of *Bacteroides* was decreased significantly (*P < *0.05). After addition of commercial fiber, compared with the LPS+AF group, the relative abundance of *Bradyrhizobium* was significantly increased (*P < *0.05), and the relative abundances of *norank_c__Cyanobacteria* and *Helicobacter* tended to increase (*P = *0.062 and *P = *0.100) (Fig. 5).

Microbial compositions of each group of piglets were further analyzed using linear discriminant analysis coupled with effect size (LEfSe). The results showed that 5 taxa in the LPS group were significantly less abundant than those in the CK group, 22 taxa in the LPS+AF group were significantly more abundant than those in the LPS group, and 20 taxa in the LPS+CF group were significantly more abundant than those in the LPS group. At the phylum level, our LEfSe analysis revealed that *Proteobacteria* was significantly enriched in the ileal mucosa of piglets in the CK group, *Tenericutes* was significantly enriched in the LPS group, *Nitrospirae* was significantly enriched in the LPS+AF group, and *Proteobacteria*, *Chloroflexi*, *Cyanobacteria*, and *Acidobacteria* were significantly enriched in the LPS+CF group. At the genus level, eight genera (*Anaerostipes*, *Bacteroides*, *norank_f__Thiotrichaceae*, *Eubacterium__fissicatena_group*, *Oscillospira*, *Devosia*, *Truepera*, and *norank_f__NS9_marine_group*) were significantly enriched in the CK group, one genus (*Mycoplasma*) was significantly enriched in the LPS group, five genera (*Campylobacter*, *norank_c__KD4_96*, *norank_f__Caldilineaceae*, *Nitrospira*, and *Sphingopyxis*) were significantly enriched in the LPS+AF group, and four genera (*Bradyrhizobium*, *Pediococcus*, *norank_c__Cyanobacteria*, and *Hyphomicrobium*) were significantly enriched in the LPS+CF group.

We also observed that the class *Betaproteobacteria* and the orders *Thiotrichales* and *Deinococcales* were enriched in CK piglets, the class *Mollicutes* and the order *Mycoplasmatales* were enriched in LPS piglets, the classes *KD4_96*, *Nitrospira*, and *Caldilineae* and the orders *Enterobacteriales*, *norank_c__KD4_96*, *Acidimicrobiales*, *Caldilineales*, and *norank_c__Nitrospira* were enriched in LPS+AF piglets, and the classes *Alphaproteobacteria*, *Bacilli*, *Cyanobacteria*, and *Acidobacteria* and the orders *Rhizobiales*, *Lactobacillales*, and *norank_c__Cyanobacteria* were enriched in LPS+CF piglets. Similarly, at the family level, *Bacteroidaceae*, *Thiotrichaceae*, *Trueperaceae*, and *NS9_marine_group* were highly enriched in the CK group, *Mycoplasmataceae* was highly enriched in the LPS group, *Enterobacteriaceae*, *Xanthobacteraceae*, *Campylobacteraceae*, *Sphingomonadaceae*, *norank_c__KD4_96*, *Comamonadaceae*, *Caldilineaceae*, and *norank_c__Nitrospira* were highly enriched in the LPS+AF group, and *Bradyrhizobiaceae*, *Lactobacillaceae*, *norank_c__Cyanobacteria*, *Hyphomicrobiaceae*, and *Streptococcaceae* were highly enriched in the LPS+CF group (Fig. S2C to H).

### Biofunction prediction of the intestinal microbial community.

In this study, PICRUSt was based on the OTU tree in the Greengenes database. The genetic information on the OTUs was used to infer the gene function spectrum of common ancestors and to infer the gene function spectrum of other untested species in the Greengenes database, allowing us to construct the full archaeal and bacterial domain spectrum of the gene function prediction spectrum and finally “map” the sequenced microbial composition into the database to achieve prediction of the metabolic function of the flora.

The predicted results can be enriched at three different levels of the KEGG pathways. The results showed that metagenome was highly regulated in response to dietary fiber. At KEGG pathway level 1, metabolic pathways were divided into six functional categories, in which metabolism was the main function of microflora (Fig. S3A). At KEGG pathway level 2, the membrane transport, carbohydrate metabolism, amino acid metabolism, replication and repair, translation, and energy metabolism changed in response to LPS and dietary fiber (Fig. S3B). At KEGG pathway level 3, the heat map showed the top 25 richest KEGG pathways, including transporters, ribosome, DNA repair and recombination proteins, and purine metabolism (Fig. S3C). The relative enrichment of different metabolic pathways in intestinal microflora can distinguish the effects of different treatments on intestinal function of piglets, so the significance test of differences between groups was carried out to observe the differences in gene function resulting from different treatments (level 3).

10.1128/mSystems.01374-20.5 FIG S3 The composition of intestinal microbial function of piglets in different groups. (A) KEGG pathway annotations in level 1. (B) KEGG pathway annotations in level 2. (C) KEGG pathway annotations in level 3. CK, basal diet (control); LPS, basal diet plus LPS; LPS+AF, basal diet plus LPS plus alfalfa fiber; LPS+CF, basal diet plus LPS plus commercial fiber. Download FIG S3, TIF file, 2.1 MB. Copyright © 2021 Sun et al. 2021 Sun et al. https://creativecommons.org/licenses/by/4.0/ This content is distributed under the terms of the Creative Commons Attribution 4.0 International license.

**FIG 6:**
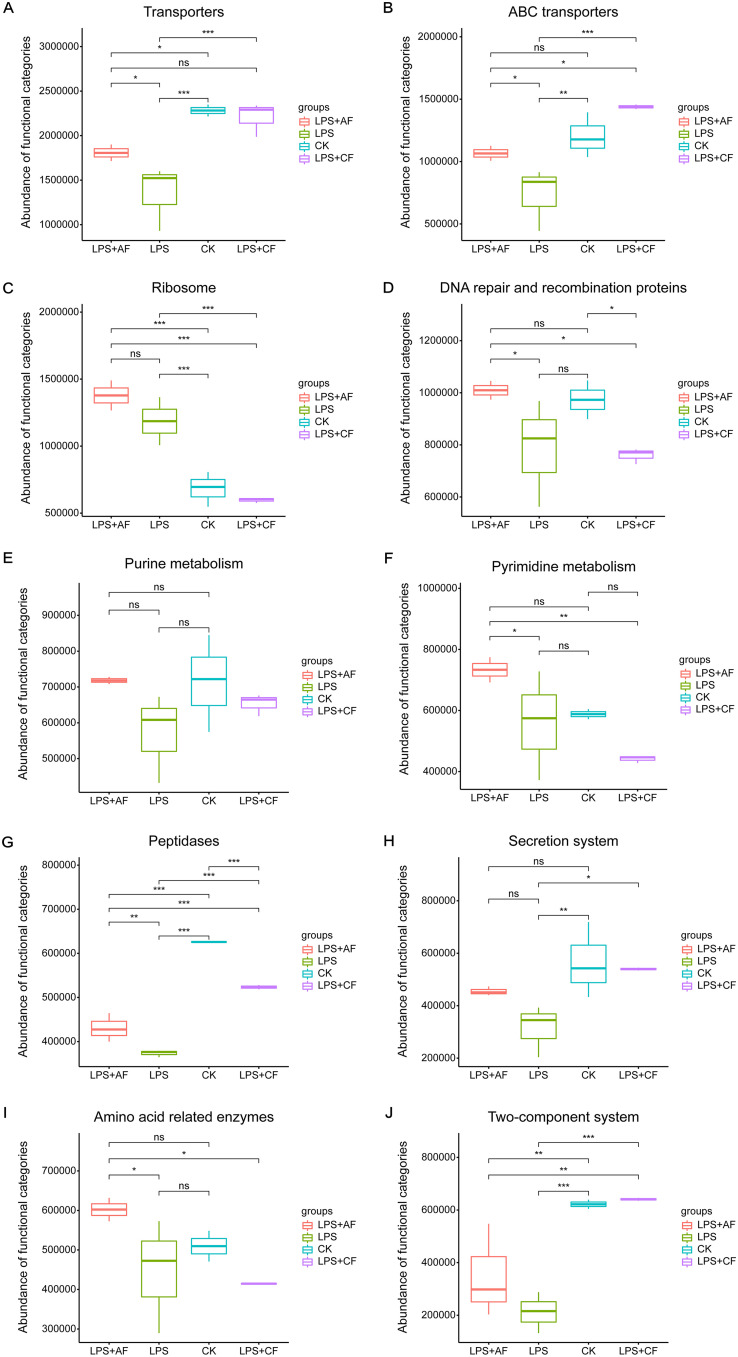
Differences in intestinal microbial function of piglets in different groups at level 3. (A) Transporters. (B) ABC transporters. (C) Ribosome. (D) DNA repair and recombination proteins. (E) Purine metabolism. (F) Pyrimidine metabolism. (G) Peptidases. (H) Secretion system. (I) Amino acid related enzymes. (J) Two-component system. The data are means ± SD. *, *P < *0.05; **, *P < *0.01, ***, *P < *0.001; ns, not significant. CK, basal diet (control); LPS, basal diet plus LPS; LPS+AF, basal diet plus LPS plus alfalfa fiber; LPS+CF, basal diet plus LPS plus commercial fiber.

In the top 10 abundance pathways, LPS stimulation significantly decreased the expression of transporters, ABC transporters, peptidases, secretion system, and two-component system genes in the intestine (*P < *0.05) had a tendency to decrease the expression of DNA repair and recombination proteins and purine metabolism genes (*P = *0.074 and *P = *0.097) and significantly increased the expression of ribosome genes (*P < *0.05). After addition of alfalfa fiber, the expression of transporters, ABC transporters, DNA repair and recombination proteins, pyrimidine metabolism, peptidases, and amino acid-related enzymes genes increased significantly (*P < *0.05), while the expression of ribosome, purine metabolism, and secretion system genes tended to increase (*P = *0.094, *P = *0.089, and *P = *0.084). After addition of commercial fiber, compared with LPS group, the expression of transporters, ABC transporters, peptidases, secretion system genes, and two-component system genes increased significantly (*P < *0.05), while the expression of ribosome gene decreased significantly (*P < *0.05). After addition of commercial fiber, compared with the LPS+AF group, the expression of ABC transporters, peptidases, and two-component system genes increased significantly (*P < *0.05), and the expression of transporter genes tended to increase (*P = *0.052), while the expression of genes for ribosomes, DNA repair and recombination proteins, pyrimidine metabolism, and amino acid-related enzymes decreased significantly (*P < *0.05) (Fig. 6).

### Correlation analysis between ileal microflora and metabolites.

**FIG 7:**
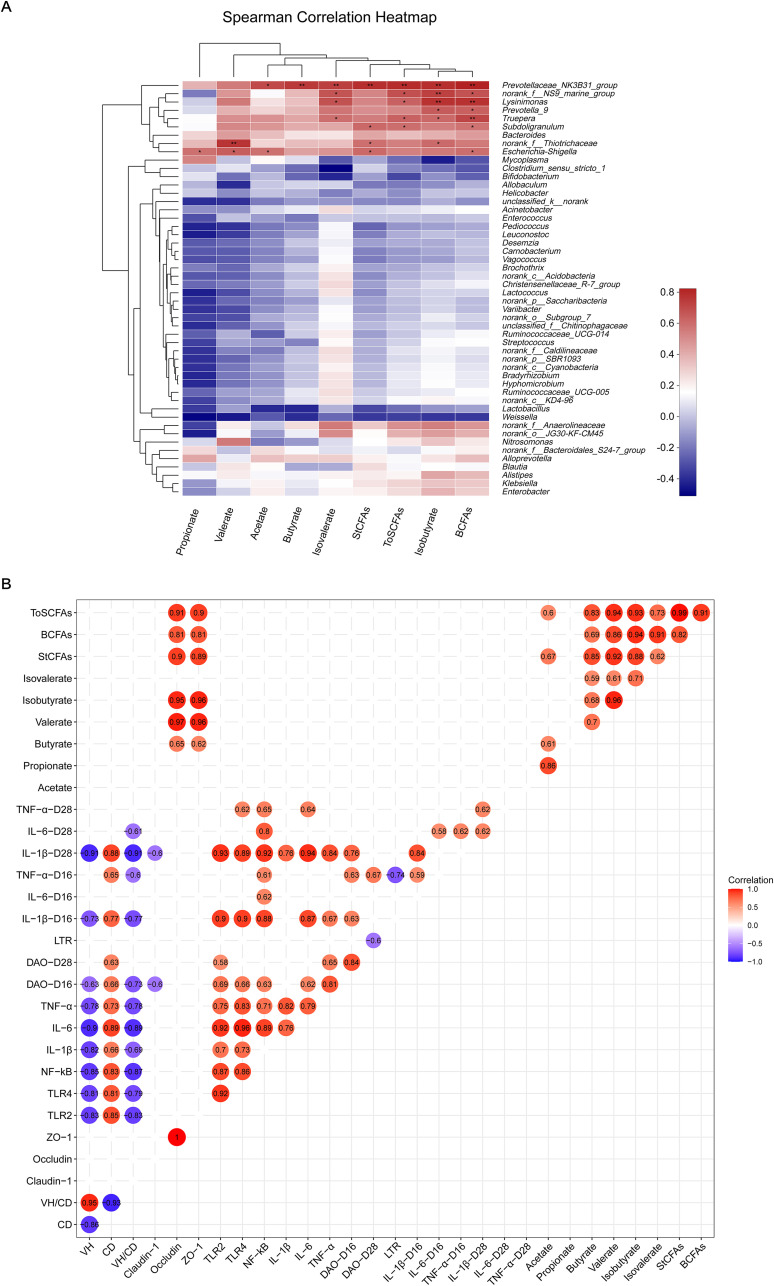
Association and model predictive analysis. (A) Analysis of the correlation between intestinal microflora and intestinal short-chain fatty acid content. (B) Analysis of the correlation between intestinal morphology, intestinal gene expression, and plasma cytokine level and intestinal short-chain fatty acid content. *, *P < *0.05; **, *P < *0.01. Red represents positive correlation, and blue represents negative correlation.

The abundance of *Prevotellaceae_NK3B31_group* was positively correlated with the levels of acetate, butyrate, isobutyrate, isovalerate, BCFAs, StCFAs, and ToSCFAs (*P < *0.05). The abundances of *norank_f__NS9_marine_group*, *Lysinimonas*, and *Truepera* were positively correlated with the levels of isobutyrate, isovalerate, BCFAs, and ToSCFAs (*P < *0.05). The abundance of *Prevotella_9* was positively correlated with the contents of isobutyrate and BCFAs (*P < *0.05). The abundance of *Subdoligranulum* was positively correlated with the contents of BCFAs, StCFAs, and ToSCFAs (*P < *0.05). The abundance of *norank_f__Thiotrichaceae* was positively correlated with the contents of valerate, isobutyrate, and StCFAs (*P < *0.05). The abundance of *Escherichia-Shigella* was positively correlated with the contents of acetate, propionate, valerate, StCFAs, and BCFAs (*P < *0.05) (Fig. 7A).

### Analysis of correlation between ileal metabolites and ileal morphology, ileal gene expression, and plasma cytokine levels.

Pearson correlation analysis was used to determine the correlation between ileal metabolites and ileal morphology, ileal gene expression, and plasma cytokine levels. The metabolic association heat map indicated that there was a positive or negative correlation between SCFAs and intestinal morphology, intestinal gene expression, and plasma cytokine levels. First, considering the correlation between intestinal morphology and the expression of intestinal inflammatory factors and the contents of plasma inflammatory factors, VH and VH:CD were negatively correlated with the expression of intestinal inflammatory factors (TLR2, TLR4, NF-κB, IL-1β, IL-6, and TNF-α) and the levels of plasma inflammatory factors (IL-1β, IL-6, and TNF-α) (*P < *0.05), while the CD was positively correlated with the expression of intestinal IL-1β and TNF-α and the level of plasma TNF-α (*P < *0.05). Second, when the correlation between intestinal inflammatory factor expression, plasma inflammatory factor levels, and intestinal and plasma barrier-related factor levels was considered, there was a significant positive correlation between intestinal inflammatory factor expression, plasma inflammatory factor contents, and plasma DAO activity (*P < *0.05). Third, considering the correlation between metabolites and intestinal barrier function, the contents of SCFAs were positively correlated with the mRNA expression of occludin and ZO-1 (*P < *0.05). Furthermore, there was a significant negative correlation between plasma TNF-α content, DAO activity, and lymphocyte transformation rate (*P < *0.05) (Fig. 7B).

## DISCUSSION

During weaning, piglets produce stress responses due to incomplete intestinal development, insufficient passive immunity, and changes in nutrition and environment, which are usually accompanied by severe growth inhibition and diarrhea (1, 3). Intraperitoneal or intravenous injection of a certain amount of lipopolysaccharide is a classical method to simulate immune stress. LPS is a membrane component of Gram-negative bacteria which can induce bacterial infection symptoms in piglets, such as anorexia, lethargy, and fever (48, 49). Liu et al. (9) found that during the first challenge (days 14 to 21), LPS stimulation significantly decreased the ADFI and ADG of piglets, and during the second challenge (days 21 to 28), LPS stimulation significantly decreased the ADG of piglets. Mao et al. (50) reported that LPS stimulation significantly reduced the ADG of piglets. In this study, LPS stimulation significantly decreased the ADG of piglets, significantly increased the F:G, and had a tendency to reduce ADFI. This is similar to the results of previous studies. After LPS stimulation, the secretion of inflammatory cytokines increases, which diverts the energy used for growth and development to maintain a highly activated immune system, thus reducing production performance (51, 52).

The present study shows that the addition of DF can increase the ADFI of piglets, lead to the increase of growth rate, and thus improve the F:G. Lindberg (53) found that the source and type of fiber determined the effect of fiber on the growth and development of the host. In this study, the addition of alfalfa fiber and commercial fiber significantly improved the growth performance of weaned piglets. This is consistent with the findings of Adams et al. (54) and Kroismayr and Roberts (55). Their studies showed that the addition of alfalfa fiber or commercial fiber could increase the ADFI and ADG of piglets. From the perspective of the water solubility of the fiber, alfalfa fiber is mainly composed of insoluble dietary fiber such as cellulose, lignin, and xylan, accounting for more than 90% of the total dietary fiber of alfalfa. Similarly, the commercial fiber used in this study is also a kind of insoluble fiber, with a total dietary fiber content of 95%, an insoluble dietary fiber content of 94%, and a lignin content of 30% (54, 56–58). The study by Schedle et al. (59) also showed that the addition of insoluble fiber (wheat bran) increased ADFI and ADG of piglets. Hermes et al. (60), Gerritsen et al. (61), and Che et al. (62) also found that the addition of dietary fiber (wheat bran and beet pulp, oat shell and wheat straw, and astragalus fiber) could improve the ADG and feed conversion rate of piglets.

The growth performance of piglets may be related to diarrhea, so the occurrence of diarrhea in piglets was recorded in this study. The diarrhea rate reflects the incidence of diarrhea in piglets, and the diarrhea index reflects the severity of diarrhea. Through the observation of these two indexes, the diarrhea status of piglets can be fully assessed. It was reported that LPS could induce severe gastroenteritis and diarrhea in young animals and infants (10, 63). In this study, LPS challenge induced diarrhea in piglets, causing growth inhibition, which was consistent with the results of previous studies (9, 10, 63). Previous studies reported that the addition of coarsely ground wheat bran significantly reduced the fecal score of piglets infected with Escherichia coli K88 (64). The addition of insoluble nonstarch polysaccharides, such as oat shell, could reduce the incidence of diarrhea in piglets (15, 65). The study by Adams et al. (54) also showed that the addition of alfalfa fiber significantly decreased the diarrhea rate of piglets. Che et al. (62) also found that the addition of astragalus fiber to the diet of piglets significantly reduced the diarrhea rate. Similar to the above results, our study also found that the addition of dietary fiber significantly reduced the diarrhea rate and diarrhea index of piglets. Therefore, adding dietary fiber can effectively prevent diarrhea in weaned piglets. In this experiment, the addition of DF improved the growth performance and the diarrhea of piglets, which may be related to the SCFAs produced by dietary fiber fermented by intestinal microorganisms. Previous studies showed that SCFAs could promote the maturation of the gastrointestinal tract, improve intestinal barrier function, and regulate body immunity, thereby reducing diarrhea rate and improve growth performance of piglets (33, 66, 67).

Our previous studies showed that under normal conditions (not attacked by LPS), dietary fiber supplementation had no effect on the growth performance of weaned piglets (56), indicating that the level of fiber in the basal diet was sufficient to maintain the growth and physiological function of weaned piglets under normal physiological conditions. However, DF could alleviate the growth retardation of weaned piglets after LPS challenge, suggesting the importance of exogenous dietary fiber supply during inflammation. Therefore, the main purpose of this experiment was to study the effect of DF on intestine-related indexes of piglets challenged with LPS but not on intestine-related indexes of non-LPS-challenged piglets.

Intestinal morphology is an important index of the health of the small intestine, including VH, CD, and VH:CD (40). It can be used to measure the integrity of small intestinal barrier, the ability to digest and absorb nutrients (68–70), immunity (71), and the number of mature intestinal epithelial cells (72–74), and it is also an important factor in determining the sensitivity of piglets to diarrhea (75). Many studies have shown that LPS challenge can lead to various morphological changes of the intestine, such as intestinal bleeding and necrosis (76), decrease of VH, and increase of CD (63, 77, 78), resulting in increased intestinal mucosal permeability and impaired barrier function (79). As expected, our results showed that LPS challenge increased the CD in the ileum, suggesting that LPS led to intestinal morphological damage. Compared with LPS only, the addition of DF increased the VH and VH:CD and decreased the CD. Similarly, Hedemann et al. (80) found that the addition of insoluble fiber (barley hull) significantly increased the VH of the small intestine. The studies by Chen et al. (67, 81) also showed that adding insoluble fiber (wheat bran) could increase the VH and VH:CD of jejunum and ileum, and improve the intestinal morphology. This is also consistent with the results of our study showing that the addition of DF improved the growth performance of piglets, indicating that DF can improve the morphology of ileal mucosal epithelium and thus improve the ability to digest and absorb nutrients to promote the growth of the body.

The intestinal barrier can prevent pathogens, toxins, and antigens from entering mucosal tissue and play an important role in maintaining the balance of the intestinal environment (82, 83). Intestinal barrier function can be evaluated by many indicators, including intestinal mucosal permeability (84, 85) and DAO activity (86, 87). Tight junctions are an important part of the intestinal mucosal mechanical barrier, which mainly includes many proteins such as claudin, occludin, ZO-1, and junction adhesion molecules (88, 89). Tight-junction proteins play an important role in maintaining the integrity of the intestinal barrier, thus preventing the spread of harmful substances, such as intestinal bacteria and other antigens, between epithelial cells (90); the decrease of tight junction protein level is usually associated with epithelial barrier dysfunction (91). LPS challenge not only affects intestinal morphology but also adversely affects intestinal barrier function (92). Consistent with the above, our results showed that LPS challenge decreased the gene expression levels of occludin and ZO-1 in ileal mucosa. Compared with LPS alone, dietary fiber supplementation increased the gene expression levels of claudin-1 and occludin, indicating that dietary fiber supplementation improved intestinal barrier function.

DAO is a highly active intracellular enzyme in intestinal epithelial cells. With the increase of intestinal permeability, it can pass through the epithelial mucosa and enter the plasma, resulting in an increase in the activity of DAO in the plasma (93). This study also found that LPS challenge increased plasma DAO activity, while DF decreased plasma DAO activity. This was consistent with the observation that DF significantly increased the expression level of intestinal tight junction protein in piglets challenged by LPS, which further indicated that DF could reduce the damage of intestinal mucosal barrier caused by LPS, effectively promote the formation of tight junction structure between cells, and then reduce intestinal mucosal permeability and enhance intestinal mucosal mechanical barrier function in early weaned piglets. Previous studies also showed that DF could reduce the plasma DAO activity and increase the mRNA expression of tight junction proteins (occludin and ZO-1) in the ileum of piglets, thus maintaining the integrity of the intestinal mucosal mechanical barrier (66, 94, 95).

Intestinal epithelial cells can provide an immune barrier for microbial invasion through congenital and adaptive immune responses (96). During the transition period of weaning, the development and function of the immune systems of piglets were impaired. Toll-like receptors (TLRs) are one of the important pattern recognition receptors in the innate immune defense system in organisms, and they play a key role in mediating the body’s inflammatory response and related signal transduction (97–99). In recent years, studies on the functions of TLR2 and TLR4 of TLR family members clearly revealed that the upregulated expression of TLR2 and TLR4 can release transcription factor NF-κB from the I-kB/NF-κB complex and transfer it into the cell, causing the release of related inflammatory factors, including IL-1β, IL-6, and TNF-α, and participating in the immune response against bacterial pathogens (100, 101). It is well known that LPS, a bacterial cell wall component that may initiate a destructive inflammatory response, is a powerful stimulator of the host’s natural defense system, and TLR2 and TLR4 have also been identified as signal receptors of bacterial cell wall components (40, 96, 102–104). Consistent with this, our study found that LPS challenge increased the mRNA expression of TLR2 and TLR4 in ileal mucosa. Compared with LPS alone, dietary fiber supplementation decreased the mRNA expression levels of TLR2, TLR4, and NF-κB. Xiao et al. (105) also found that the addition of chitosan could reduce the gene expression level of TLR4 in the jejunum of piglets.

Lymphocyte transformation rate (proliferation) is an important index for evaluating cellular immune function (9). Previous studies showed that LPS stimulation significantly decreased the number of T lymphocytes in the ileum of piglets (63). In our study, the lymphocyte transformation rate of peripheral blood decreased significantly after LPS stimulation, while the addition of DF increased the lymphocyte transformation rate of peripheral blood and enhanced the cellular immune function of immune stress piglets. Mao et al. (50) also found that the addition of β-glucan increased the peripheral blood lymphocyte transformation rate of piglets challenged with LPS. These results suggest that DF may enhance the host’s disease resistance by inhibiting the activation of NF-κB and regulating the body’s immunity.

We further speculated whether DF may have a beneficial effect on the intestinal tract by alleviating intestinal inflammation. Previous studies showed that the intestinal tight junction barrier was also dynamically regulated by cytokines (106) and that most proinflammatory cytokines, such as IL-1β, IL-6, and TNF-α, play a key role in immune stress, which could increase intestinal epithelial permeability, induce pathological opening of intestinal tight junction barriers (107), and mediate systemic effects of inflammation, thus changing animal behavior, metabolism, and neuroendocrine function and ultimately inhibiting growth (48, 108). It is well known that LPS causes intestinal barrier dysfunction and induces intestinal inflammation (92, 109). This study also found that LPS challenge increased the gene expression level of IL-6 in ileal mucosa. Compared with LPS alone, dietary fiber supplementation decreased the mRNA expression levels of IL-1β, IL-6, and TNF-α. Similar to this study, Shang et al. (95) and Chen et al. (66) also found that the addition of insoluble fiber (wheat bran or lignocellulose) reduced the gene expression levels of IL-1β and TNF-α in the ileum. Therefore, the improvement of intestinal barrier function by DF may be related to its downregulation of IL-1β, IL-6, and TNF-α gene expression levels.

It was also found that LPS significantly increased the concentrations of IL-1β and TNF-α in plasma of piglets, which may indicate that LPS can promote the synthesis and release of inflammatory cytokines such as IL-1β and TNF-α by activated immune cells. Yi et al. (110), Wang et al. (111), and Zhang et al. (98) also found that LPS challenge significantly increased the concentrations of IL-1β, IL-6, and TNF-α in the blood of piglets. Compared with the LPS group, the addition of DF reduced the concentrations of IL-1β, IL-6, and TNF-α. The studies of Pouillart et al. (112), Shang et al. (95), and Che et al. (62) also showed that adding dietary fiber (dextrin fiber, wheat bran, and astragalus fiber) could reduce the concentrations of IL-1β, IL-6, and TNF-α in blood of piglets. Therefore, DF may exert an anti-inflammatory effect by regulating the expression levels and concentrations of proinflammatory cytokines.

In summary, these results suggest that dietary fiber supplementation can reduce the damage to intestinal morphology and barrier function caused by LPS. The protective effect of DF on the intestine may be closely related to its inhibition of TLR signal pathways, reduction of intestinal proinflammatory cytokine expression, and improvement of immune function of piglets.

In addition to the complete intestinal barrier, the best intestinal flora can also prevent pathogen attachment and maintain the intestinal function and health of the host (96, 113, 114). We have also observed microbial changes in the ileum of piglets, which is consistent with previous studies (45, 96). Complex and large microbial communities colonize the intestines of mammals, and the intestinal microflora of humans and animals plays an important role in nutrient digestion, intestinal morphology, immune development, and regulation of host gene expression (115–118). Imbalance of the microbiota can affect the host’s innate and adaptive immune system through microbial cellular composition and metabolite signals (96). According to our current research, DF significantly changed the ileal microflora of piglets stimulated by LPS, increased the abundance of members of the microflora related to carbohydrate metabolism, and reduced the number of proinflammatory bacteria.

At the phylum level, we found that the addition of DF increased the relative abundances of *Proteobacteria*, *Chloroflexi*, *Cyanobacteria*, *Acidobacteria*, *Planctomycetes*, *Nitrospirae*, *Spirochaeta*, *Gemmatimonadetes*, and *Fusobacteria*. Previous studies also found that the relative abundances of *Chloroflexi*, *Acidobacteria*, *Nitrospirae*, *Gemmatimonadetes*, and *Cyanobacteria* in the ileum of piglets were increased, improving the growth performance of piglets, which may be related to reducing the body’s oxidative stress and alleviating inflammation (119, 120). Our study also found that the addition of DF alleviated the inflammatory reaction of piglets and increased the relative abundances of *Chloroflexi*, *Acidobacteria*, *Nitrospirae*, *Gemmatimonadetes*, and *Cyanobacteria*. Pan et al. (121) found that the addition of xylo-oligosaccharides significantly increased the relative abundance of *Planctomycetes* in the intestinal contents of growing pigs and increased the concentrations of acetate, straight-chain fatty acids, and total SCFAs. This is consistent with the increase in the concentrations of acetate, straight-chain fatty acids, and total SCFAs and the increase in the relative abundance of *Planctomycetes* after the addition of DF in this study.

Kim et al. (122) showed that the number of *Spirochaeta* in the jejunum of piglets increased, which promoted the growth of piglets. Xu et al. (123) found that pectin-rich diets increased the relative abundance of *Spirochaeta* in the intestinal contents of piglets, which may be related to the fact that *Spirochetes* can produce butyrate to promote intestinal development and health (124, 125). The results of this study also showed that DF increased the number of *Spirochaeta* and the concentration of butyrate in the ileum of piglets. Xu and Zhang (126) also found that the addition of lentinan increased the relative abundances of *Proteobacteria*, *Chloroflexi*, *Gemmatimonadetes*, *Nitrospirae*, and *Planctomycetes* in the small intestines of mice and played a role in regulating immunity and anti-inflammation. Another study found that the number of *Chloroflexi*, *Proteobacteria*, *Gemmatimonadetes*, and *Acidobacteria* in the intestines of rats increased, which reduced the colitis induced by dextran sodium sulfate (DSS) (127). Similar to the above results, our study also found that DF increased the number of *Nitrospirae*, *Planctomycetes*, *Chloroflexi*, *Proteobacteria*, *Gemmatimonadetes*, and *Acidobacteria* in the ileum of piglets, and alleviated the intestinal inflammation induced by LPS.

Previous studies also found that there was a correlation between mucosal gene expression levels and SCFA concentration. Butyrate-producing bacteria (*Fusobacteria*) were positively correlated with tight junction protein (ZO-1) expression levels. Acetate, propionate, caproate, and branched-chain fatty acids could stimulate the expression of short-chain fatty acid receptors (free fatty acid receptor 2 [FFAR2] and FFAR3) and barrier-related genes (mucin 4 [MUC4]) (124, 128, 129). In our study, DF increased the relative abundance of *Fusobacteria* and improved the intestinal barrier function, which may be related to the increase of the concentrations of acetate, propionate, butyrate, and branched-chain fatty acids. The study of Xu et al. (123) also showed that the addition of DF (inulin or pectin) increased the number of butyrate-producing bacteria (*Fusobacteria* and *Proteobacteria*) in the intestinal contents of piglets and also increased the expression levels of tight junction proteins (ZO-1 and occludin) (124, 128).

At the genus level, we found that the addition of DF increased the relative abundances of *Bradyrhizobium*, *Pediococcus*, *norank_c__Cyanobacteria*, and *Streptococcus* and decreased the relative abundance of *Bacteroides*. Previous studies found that enzymes involved in butyrate synthesis, such as butyryl coenzyme A (butyryl-CoA) dehydrogenase, butyryl-CoA transferase, and butyrate kinase, were widespread in the intestinal microbiota. Members of the *Actinobacteria*, *Fusobacteria*, *Proteobacteria*, *Spirochaetes*, and *Thermotogae* have also been identified as potential butyrate-producing bacteria (124), while *Bradyrhizobium* belongs to the family *Bradyrhizobiaceae* of the phylum *Proteobacteria*. This is consistent with results in the present study showing that the relative abundance of *Bradyrhizobium* in the ileum of piglets was increased after the addition of DF, which then increased the concentration of butyrate.

*Pediococcus* belongs to the family *Lactobacillaceae* of phylum *Firmicutes*, which can ferment DF to produce propionate (33, 130) through the succinate pathway, reduce the production of TNF-α, and inhibit the activity of NF-κB, thus alleviating the inflammatory response induced by LPS (131). The SCFA results of our study also showed that the concentration of propionate in intestinal contents increased after the addition of DF, which was consistent with the increase in the relative abundance of *Pediococcus*. Maier et al. (132) also found that a highly resistant starch diet as a source of DF led to an increase in the ratio of *Firmicutes* to *Bacteroides* and the abundance of some specific members of *Firmicutes* in the human intestine, as well as the contents of butyrate and propionate in feces. Xu et al. (123) showed that the addition of DF (inulin and pectin) increased the relative abundances of *Cyanobacteria* and *Streptococcus* in intestinal contents.

Previous studies also showed that butyrate could be synthesized from acetate and lactate through the butyryl kinase and acetate-CoA transferase pathway (130, 133), while *Streptococcus* could produce acetate through the Wood-Ljungdahl pathway, which in turn could be interacted with by other bacteria to produce butyrate (33, 130). The results of this study showed that the concentrations of acetate and butyrate in the ilea of piglets were increased after the addition of DF, which was also consistent with the increase in the relative abundance of *Streptococcus*. Previous studies also found that the potential pathogen *Bacteroides* was associated with poor intestinal health and was often very abundant in patients with colitis. The decrease of its abundance can reduce the severity of colitis (127, 134–136), and the increase of its abundance is usually accompanied by diarrhea (137). Our study also found that a decrease of the number of *Bacteroides* organisms in intestinal mucosa was accompanied by the remission of intestinal inflammation and the decrease of diarrhea rate. In addition, it has been found that the addition of DF (wheat bran, inulin, and alfalfa fiber) increased the abundances of *Firmicutes* and *Bifidobacterium* and decreased the abundance of *Bacteroides* (54, 64, 123), while obese people had higher levels of *Firmicutes* and lower levels of *Bacteroides* (138). In this study, the addition of DF increased the ADG of piglets, which was consistent with the increase of the abundances of some members of *Firmicutes* and the decrease of the abundances of some members of *Bacteroides* in the intestinal tract.

SCFAs are organic fatty acids with fewer than 6 carbon atoms in the carbon chain and include formate, acetate, propionate, butyrate, isobutyrate, valerate, isovalerate, caproate, and isocaproate, mainly acetate, propionate, and butyrate. They are mainly produced by intestinal microorganisms fermenting dietary fiber, resistant starch, oligosaccharides, and other indigestible sugars (139, 140). SCFAs are the main energy source of intestinal epithelial cells, which can maintain intestinal cell function, regulate immune response, and alleviate various inflammatory diseases. They are also an important link connecting intestinal cell metabolism, microflora, and gene expression regulation (33, 141, 142). In this study, dietary fiber supplementation increased the concentrations of SCFAs such as acetate, propionate, and butyrate in intestinal contents, which may help to improve intestinal integrity and reduce the expression of proinflammatory cytokine genes. Previous studies also confirmed this result (45, 56, 62, 66). It can be seen that the effect of DF on intestinal microflora of piglets is closely related to the changes of SCFA composition, which is beneficial to the improvement of intestinal function and growth performance of the host by improving the composition of intestinal microflora and its metabolites.

In this study, PICRUSt analysis showed that different treatments changed the gene function of several microorganisms. Carbohydrate metabolism was the primary function in all groups of piglets, which may be because the main components of feed and the type of substrate fermented by dominant microorganisms were carbohydrates. Previous studies showed that intestinal microflora of piglets with diarrhea was characterized by reduced gene expression related to transporters, ABC transporters, DNA repair and recombinant proteins, purine metabolism, pyrimidine metabolism, and secretory systems (143). This study also found that after LPS stimulation, the diarrhea rate of piglets increased and the gene expression of transporters, ABC transporters, DNA repair and recombinant proteins, purine metabolism, peptidases, secretory systems, and two-component systems decreased. This may be related to the functions of these KEGG pathways; for example, the ABC transporters are primary transporters that couple the energy stored in ATP to the movement of molecules across the membrane, which are linked with multidrug resistance in both bacteria and eukaryotes (144). A general overview of human DNA damage response pathways also indicated that deficient DNA repair may affect genomic stability and induce tumorigenesis (145). The addition of DF could increase the gene expression of transporters, ABC transporters, DNA repair and recombinant proteins, purine metabolism, pyrimidine metabolism, peptidases, secretory systems, amino acid-related enzymes, and two-component systems. Therefore, dietary fiber supplementation in the diet may help to regulate the abnormal function of intestinal microflora caused by stress and thus prevent the occurrence of diseases during weaning.

In summary, these results indicate that dietary fiber supplementation can regulate the host intestinal microflora, increase the abundance of anti-inflammatory bacteria and SCFA-producing bacteria, and increase the concentration of SCFAs, which may help explain the role of DF in reducing intestinal inflammation and injury caused by LPS, thereby maintaining intestinal health and normal growth and development of the body.

The correlation analysis between intestinal microflora and SCFAs showed that *Prevotellaceae_NK3B31_group* and *Prevotella_9* were significantly positively correlated with the concentrations of acetate, butyrate, isobutyrate, isovalerate, straight-chain fatty acids, branched-chain fatty acids, and total short-chain fatty acids. Previous studies also found that *Prevotella* contributed to the degradation of plant carbohydrates (146), which could produce acetate through pyruvate via the acetyl-CoA pathway and further synthesize butyrate by other bacteria using acetate as the substrate through the butyryl kinase and acetate-CoA transferase pathways (33, 130, 133). In addition, in our study, the content of branched-chain fatty acids was significantly positively correlated with *Prevotellaceae_NK3B31_group*, *norank_f__NS9_marine_group*, *Lysinimonas*, *Truepera*, and *Prevotella_9*. Some studies have shown that branched-chain fatty acids can inhibit the mRNA expression of IL-8 in intestinal epithelial cells induced by LPS and alleviate the inflammation response (147), which is consistent with the increase of BCFA content in intestinal contents and downregulation of expression of intestinal mucosal inflammatory factors in piglets after the addition of DF in this study. The analysis of the correlation between the concentration of SCFAs in intestinal contents and intestinal morphology, intestinal gene expression, and plasma cytokine levels showed that the expression levels of occludin and ZO-1 were significantly positively correlated with the concentrations of butyrate, valerate, and isobutyrate. Previous studies also showed that butyrate could regulate the energy state of cells, increase the synthesis of intestinal tight junction protein, and alleviate the damage of barrier integrity caused by LPS stimulation (148). Ma et al. (149) and Peng et al. (150) also showed that butyrate could increase the expression of intestinal tight junction proteins (occludin and ZO-1), improve the tight junction between cells, inhibit intestinal permeability, and then enhance intestinal barrier function. Wang et al. (10) also found that the addition of lentinan increased the concentrations of propionate, butyrate, isobutyrate, and isovalerate in intestinal contents of piglets challenged with LPS and improved intestinal morphology and barrier function.

Through the correlation analysis of growth performance, intestinal morphology, intestinal gene expression, plasma cytokine level, intestinal microflora, and SCFAs of piglets stimulated by LPS, we concluded that LPS challenge caused the increase of blood DAO activity, intestinal microflora disorder, decrease of SCFA production, and upregulation of proinflammatory cytokine gene expression, which led to local inflammation of the intestinal tract, destruction of intestinal microbial barrier, the increase of intestinal permeability, and the increase of the secretion of blood inflammatory factors, leading to systemic inflammation and ultimately reducing the growth performance of piglets. The addition of DF can reverse this process. Specifically, DF can regulate the structure of intestinal microflora, increase the relative abundance of SCFA-producing bacteria and the content of SCFAs, and then reduce the secretion of blood proinflammatory cytokines and the gene expression levels of intestinal proinflammatory factors, thus improving intestinal morphology, maintaining intestinal barrier function, alleviating the inflammatory reaction, and eventually improving intestinal health and growth performance of piglets.

**FIG 8:**
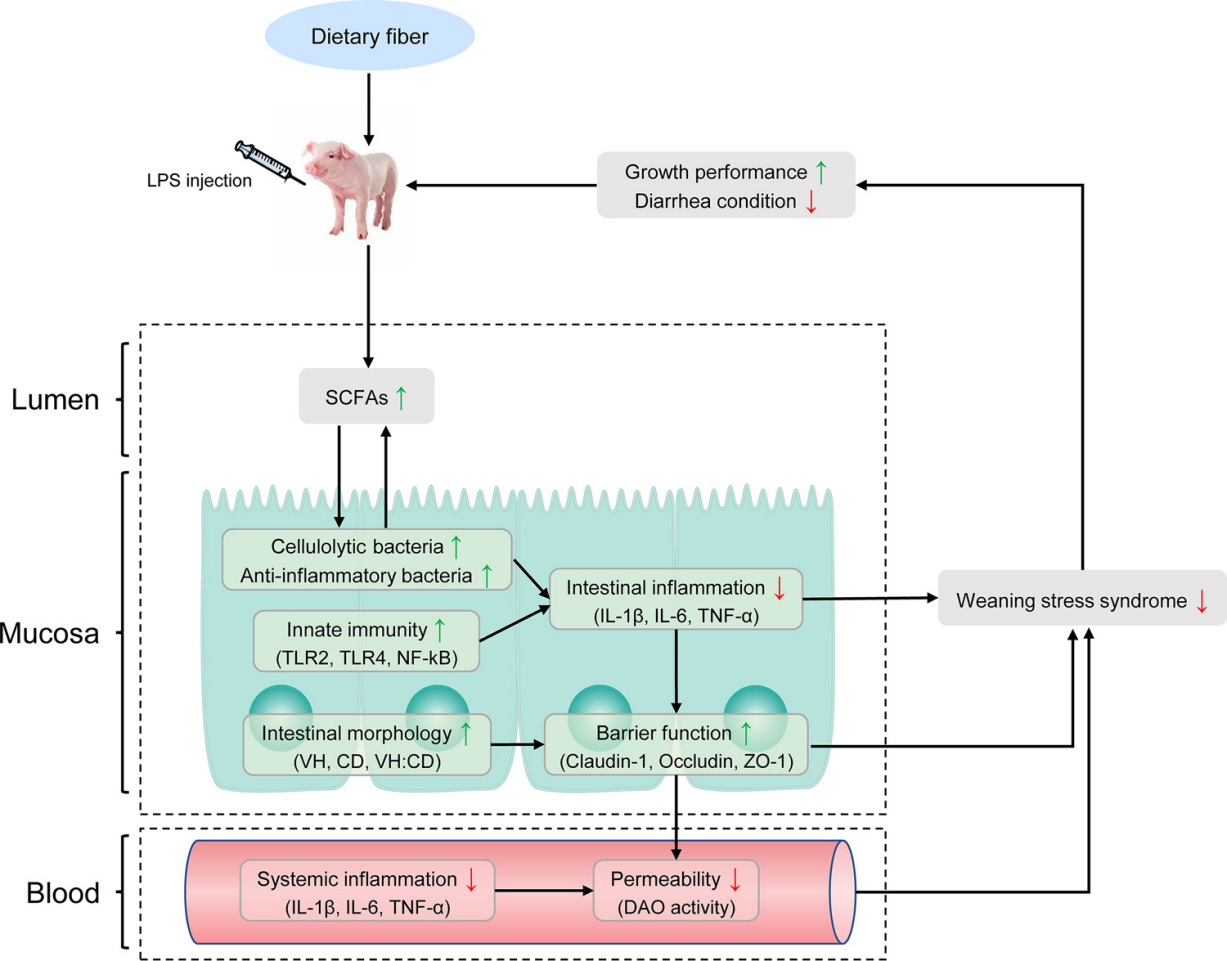
Systematic analysis of the effects of dietary fiber on growth performance, inflammatory index, intestinal barrier function, intestinal microflora, and intestinal short-chain fatty acid content in piglets challenged by LPS.

To summarize, our study found that adding dietary fiber to the diet of LPS-challenged piglets could alleviate intestinal and systemic inflammation, improve intestinal barrier function, and ultimately alleviate the growth retardation of piglets (Fig. 8). In addition, the addition of dietary fiber significantly increased the relative abundance of SCFA-producing bacteria and the production of SCFAs. We believe that the improvement of growth performance of piglets challenged with LPS can be attributed to the beneficial effects of dietary fiber on intestinal microflora and SCFAs, thus reducing the inflammatory response of piglets, improving intestinal barrier function, and enhancing body health. These research results provide a theoretical basis and guidance for the use of specific fiber sources in the diet to improve intestinal health and growth performance of piglets and alleviate weaning stress. Our data also provide insights for studying the role of dietary fiber in regulating gastrointestinal function in human infants.

## MATERIALS AND METHODS

### Ethical approval.

The experimental protocol and procedures were approved by the Animal Care and Use Committee of Henan Agricultural University. All animal treatments and experiments were performed according to the recommendations of the guidelines for ethical review of animal welfare in the national standards of the People’s Republic of China (151).

### Animals, diets, and experimental design.

According to the principle of similar body weight, 96 Duroc × Landrace × Large White piglets with good health and similar physical condition [(7.93 ± 0.03) kg] were randomly divided into 4 groups: basal diet (control; CK), basal diet plus LPS (LPS), basal diet plus LPS plus alfalfa fiber (LPS+AF), and basal diet plus LPS plus commercial fiber (LPS+CF). There were 3 replicates in each group and 8 piglets in each replicate. The experimental period was 28 days. On the 16th and 28th days of the experiment, piglets in the LPS group, LPS+AF group, and LPS+CF group were injected intraperitoneally with LPS (LPS dissolved in normal saline; the content of LPS in injection was 500 μg/ml, and it was injected at 0.2 ml/kg of body weight (BW), which was equivalent to 100 μg/kg of BW) (78, 109), the piglets in the CK group were injected with the same amount of normal saline intraperitoneally. Commercial fiber was purchased from Agromed (Austria), which contains 85% total dietary fiber and 59% crude fiber (57). LPS (Escherichia coli serotype O55:B5) was purchased from Sigma. During the whole test period, piglets ate and drank freely. The basic diet was prepared with reference to the nutritional requirements of weaned piglets (152). To prepare diets with different fiber sources, 5% alfalfa fiber or 2% commercial fiber was added to the basic diet. Dietary composition and nutrition level are shown in Table S1.

10.1128/mSystems.01374-20.1 TABLE S1 Feed composition and nutrients offered to piglets during the study. Download Table S1, DOCX file, 0.01 MB. Copyright © 2021 Sun et al. 2021 Sun et al. https://creativecommons.org/licenses/by/4.0/ This content is distributed under the terms of the Creative Commons Attribution 4.0 International license.

### Sample collection.

Four hours after injection of LPS or normal saline on the 16th and 28th days of the experiment, 1 piglet was randomly selected from each replicate of each treatment to collect 10 ml of blood from the anterior vena cava using an anticoagulation vacuum blood collection tube. Plasma was isolated by centrifugation at 3,000 rpm at 4°C for 10 min, and frozen at −80°C until subsequent analysis. After blood was collected on the 28th day, the piglets were anesthetized by intravenous injection of pentobarbital sodium (80 mg/kg of BW) and slaughtered. After the piglets were slaughtered, an ileal segment of 3 cm was preserved in 4% paraformaldehyde for routine morphological measurement. Ileal mucosa was collected to detect microflora and the expression level of related genes, and ileal contents were collected to detect microbial metabolites. All samples were collected within 15 min after slaughtering. It has been reported that LPS causes serious damage to intestinal structure and barrier function within 2 to 6 h after injection, so the experimental analysis was carried out at 4 h after LPS or normal saline injection (40, 78, 109).

### Growth performance.

During the experiment, the daily feed intake of each replicate was accurately recorded, and the health status of piglets was observed. The piglets were weighed in units of replicate on an empty stomach on the mornings of the 1st, 16th, 23rd, and 28th days of the experiment, respectively. The average daily feed intake (ADFI), average daily gain (ADG), and feed-to-gain ratio (F:G) for each stage and over the whole period of the experiment were calculated.

### Diarrhea.

After injection of LPS on the 16th day, the feces of piglets in each group were observed and recorded twice a day, in the morning and evening, within 1 week after injection and scored (0, normal; 1, sticky; 2, semiliquid; 3, liquid), and diarrhea index was calculated as described by Mao et al. (75): (∑ fecal score of piglets within 1 week after injection)/total number of piglets. When the fecal score was ≥2, diarrhea was recorded, and the diarrhea rate (as a percentage) of each group of piglets was calculated according to the formula reported by Long et al. (153): (number of piglets with diarrhea × days of diarrhea)/(number of total piglets × days of experiment) × 100.

### Blood index. (i) Levels of inflammatory factors.

The levels of inflammatory factors were determined with an enzyme-linked immunosorbent assay (ELISA) kit from Shanghai Enzyme Union Biotechnology Co., Ltd., and the operation was carried out according to the instructions.

### (ii) Levels of barrier factors.

The activity of diamine oxidase (DAO) was determined with a kit from Nanjing Jiancheng Institute of Biological Engineering, and the operation was carried out according to the instructions.

### (iii) Levels of immune factors.

The lymphocyte transformation rate (LTR) (proliferation) in peripheral blood was determined by using the Cell Counting Kit-8 (CCK-8) from Japanese Dojindo Chemical Co., Ltd. (154). First, 4 ml of lymphocyte separation solution was pipetted into a 10-ml centrifuge tube; then a 3-ml blood sample was slowly added along the tube wall, and the tube was centrifuged at 2,500 rpm for 30 min. About 1 ml of the intermediate leukocyte layer was added to 3 to 5 times the volume of RPMI 1640 culture solution for 3 washes and centrifuged at 2,000 rpm for 10 min each time, and the supernatant was discarded. Then the cells were suspended in RPMI 1640 culture medium and stained with trypan blue. The number of living cells was counted (>95%), and the cell concentration was adjusted to 2 × 10^6^/ml. A cell suspension of RPMI 1640 complete culture medium containing concanavalin A (ConA) (final concentration of 5 μg/ml) or without ConA was added to a 96-well culture plate with 100 μl per well and 3 repeats per sample. The suspensions were cultured at 5% CO_2_ and 37°C for 72 h. Four hours before the end of culture, 10 μl of 5 mg/ml CCK-8 was added to each well, and culturing was continued for 4 h. After the end of culture period, 100 μl 10% SDS–0.04 mol/liter HCl solution was added to each well. After 30 min, the light absorption value (optical density [OD] value) of each well was measured with an enzyme labeling instrument at a 450-nm wavelength. Lymphocyte transformation rate was expressed as the stimulation index (SI): OD_450_ of the well with ConA stimulation/OD_450_ of the control well.

### Intestinal morphological analysis.

After 24 h of fixation, the intestinal segment was dehydrated with gradient alcohol, made transparent with xylene, and embedded in paraffin. Three cross sections (4 μm thick) of each intestinal specimen were taken to prepare histological sections and stained with hematoxylin and eosin. Ten well-oriented and intact villi and their associated crypts were taken from each segment; the villus height (VH) and crypt depth (CD) were measured, and the ratio of villus height to crypt depth (VH:CD) was calculated by dividing villus height by crypt depth (78).

### Real-time PCR analysis.

The animal tissue RNA extraction kit (Shanghai Shenggong Bioengineering Co., Ltd.) was used according to the manufacturer’s instructions to extract total RNA from the ileum. After the purity and integrity of RNA were tested, cDNA was prepared with a PrimeScript RT kit (Toyobo) with gDNA Eraser for quantitative real-time PCR (qRT-PCR). With the GAPDH gene as an internal reference gene, the relative expression of mRNA of tight junction protein genes (claudin-1, occludin, ZO-1), Toll-like receptor-related genes (TLR2, TLR4, and NF-κB), and inflammatory cytokine genes (IL-1β, IL-6, and TNF-α) was detected by qRT-PCR. The primer sequences (Table S2) were synthesized by Shangya Biotechnology Co., Ltd. qRT-PCR was carried out according to the manufacturer’s instructions on a LightCycler 96 real-time PCR instrument (Roche) using a SYBR PreMix ExTaq qPCR kit (Tli RNase H Plus; Biosharp). The amplification procedure was 95°C for 30 s, followed by 40 cycles of 95°C for 5 s and 60°C for 30 s. The expression of related genes was analyzed by the 2^−ΔΔ^*^CT^* method (155).

10.1128/mSystems.01374-20.2 TABLE S2 Primer sequences of the studied genes. Download Table S2, DOCX file, 0.01 MB. Copyright © 2021 Sun et al. 2021 Sun et al. https://creativecommons.org/licenses/by/4.0/ This content is distributed under the terms of the Creative Commons Attribution 4.0 International license.

### DNA extraction, PCR amplification, and 16S rRNA gene sequencing.

Microbial DNA was extracted from intestinal mucosal samples using an E.Z.N.A. stool DNA kit (Omega Bio-tek, Norcross, GA, USA), according to the manufacturer’s protocols. The final DNA concentration and purity were determined using a NanoDrop 2000 UV–visible-spectrum (Vis) spectrophotometer (Thermo Scientific, Wilmington, DE, USA), and DNA quality was checked by 1% agarose gel electrophoresis. The V3-V4 hypervariable regions of the bacterial 16S rRNA gene were amplified using the primers 338F (5′-ACTCCTACGGGAGGCAGCAG-3′) and 806R (5′-GGACTACHVGGGTWTCTAAT-3′) by PCR (GeneAmp 9700; ABI, USA) (10), with the following program: 3 min denaturation at 95°C; 27 cycles of 30 s at 95°C, 30 s annealing at 55°C, and 45 s elongation at 72°C; and a final extension at 72°C for 10 min. PCRs were performed in triplicate, with each 20-μl reaction mixture containing 4 μl of 5× FastPfu buffer, 2 μl of 2.5 mM deoxynucleoside triphosphates (dNTPs), 0.8 μl of each primer (5 μM), 0.4 μl FastPfu polymerase, and 10 ng template DNA. The resulting PCR products were extracted from 2% agarose gels, further purified using an AxyPrep DNA gel extraction kit (Axygen Biosciences, Union City, CA, USA), and quantified using a QuantiFluor-ST instrument (Promega, USA), according to the manufacturer’s protocol. Purified amplicons were pooled in equimolar amounts and subjected to paired-end sequencing (2 × 300 bp) on an Illumina MiSeq platform (Illumina, San Diego, CA, USA), according to standard protocols, by Majorbio Bio-Pharm Technology Co. Ltd. (Shanghai, China) (156).

### Bioinformatics analysis of sequencing data.

Paired-end reads were merged using FLASH (157), which was designed to merge paired-end reads when at least some of the reads overlap the read generated from the opposite end of the same DNA fragment, and the splicing sequences were called raw tags. Quality filtering on the raw tags was performed under specific filtering conditions to obtain the high-quality clean tags according to the QIIME quality control process (158). The tags were compared with the reference database using the UCHIME algorithm to detect chimera sequences, which were later removed (159, 160). OTUs were clustered with a 97% similarity cutoff using UPARSE (161), and chimeric sequences were identified and removed using UCHIME. The taxonomy of each 16S rRNA gene sequence was analyzed by RDP Classifier against the SILVA (SSU132) 16S rRNA database using a confidence threshold of 70% (162, 163). Sequences with more than 97% similarity were assigned to the same OTU. A representative sequence for each OTU was screened for further annotation. OTU abundance information was normalized using a standard of sequence number corresponding to the sample with the fewest sequences. These data were analyzed on the free online Majorbio i-Sanger cloud platform (www.i-sanger.com). Venn diagrams, which visually display the numbers of common and unique OTUs among groups, were drawn by the package VennDiagram of R (v3.1.1) software. Ace index, Chao index, and Shannon index, which reflect alpha diversity, were calculated by Mothur (v1.31.2), and the corresponding rarefaction curves were drawn by R (v3.1.1) software (164). Analysis of molecular variance (AMOVA) (164) was performed to compare the different treatments. The linear discriminant analysis coupled with effect size (LEfSe) was performed to determine which microbes had a significant effect on the division of the sample (165). To probe the microbial metabolism and predict metagenome functional content from the marker genes, PICRUSt was used to explore differences in the KEGG pathway between groups (166).

### Microbial metabolites.

As described by Liu et al. (56), gas chromatography (GC) was used to determine the level of SCFAs in ileal contents. The samples were analyzed on an HP-88 column (100 m long, 0.25-mm diameter, 0.2-μm film thickness from the producer) and separated using a Trace 1310 GC with a flame ionization detector (FID). The program temperature was 70°C for 1 min, increased to 180°C at 25°C/min and held for 1 min, increased to 200°C at 10°C/min and held for 1 min, increased to 220°C at 2°C/min and held for 10 min, and finally raised to 240°C at 20°C/min and held for 6 min. The sample was run with a split ratio of 20:1 and a column flow of 1.3 ml/min. Hydrogen was used as a carrier gas. The injector temperature was 270°C, and the detector temperature was 290°C.

### Statistical analysis.

The data were analyzed by one-way analysis of variance (ANOVA) with SPSS 23.0 software (IBM, New York, NY, USA) and are expressed as means and standard deviations (SD). The differences between means were assessed using least-significant-difference (LSD) multiple-comparison tests. Differences were considered significant when *P* values were <0.05, and *P* values of >0.05 and ≤0.10 were considered to indicate a tendency.

### Data availability.

The raw reads were deposited into the NCBI Sequence Read Archive (SRA) database (accession number PRJNA687832).
