# Supplementary figures and images for: Dietary Fiber Ameliorates Lipopolysaccharide-Induced Intestinal Barrier Function Damage in Piglets by Modulation of Intestinal Microbiome
Source: mSystems. 2021 Apr 6;6(2):e01374-20. doi: 10.1128/mSystems.01374-20 (PMC8547013; doi:10.1128/mSystems.01374-20)

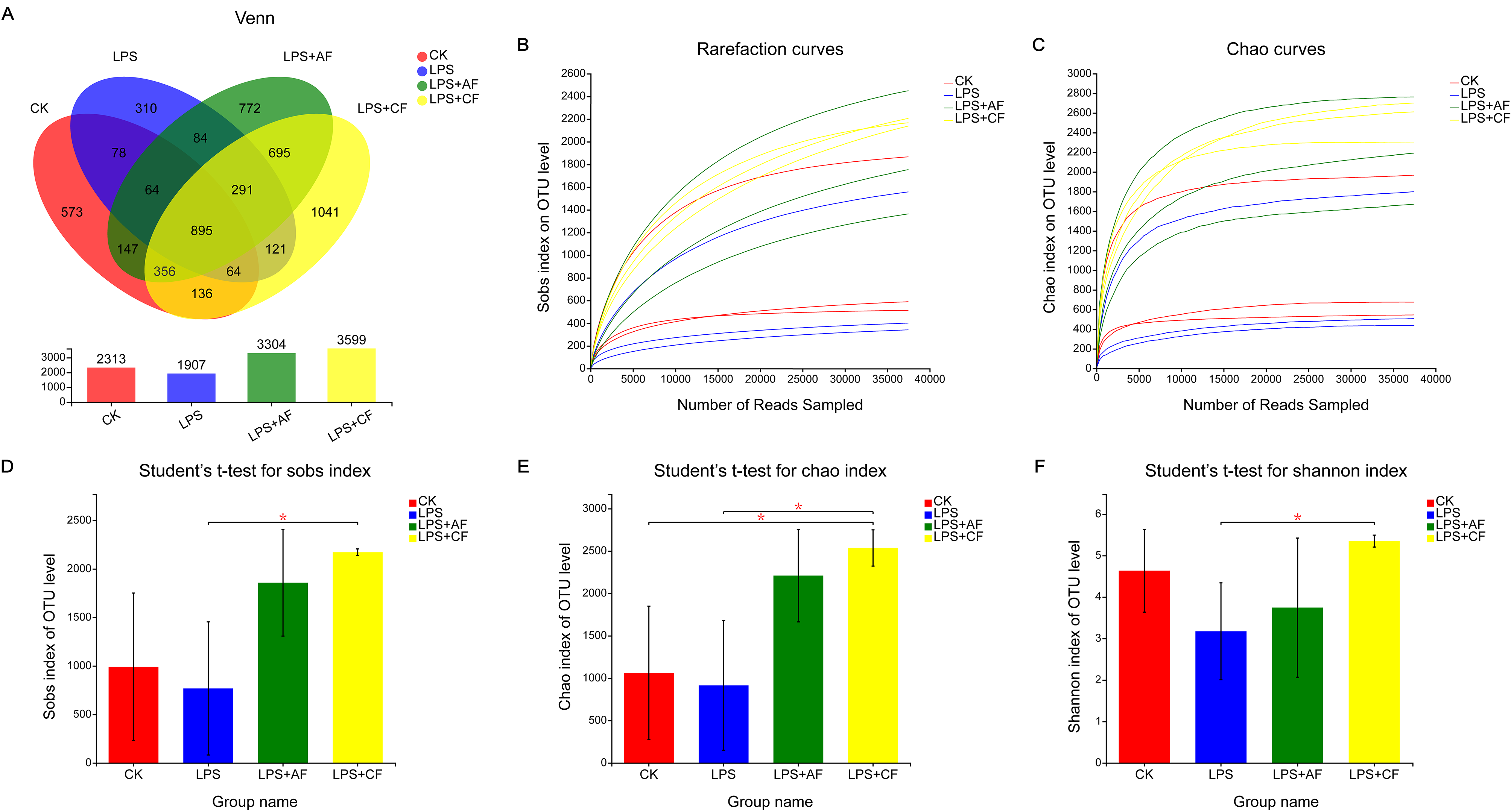

Supplement: FIG S1 [file msystems.01374-20_sf001.tif]

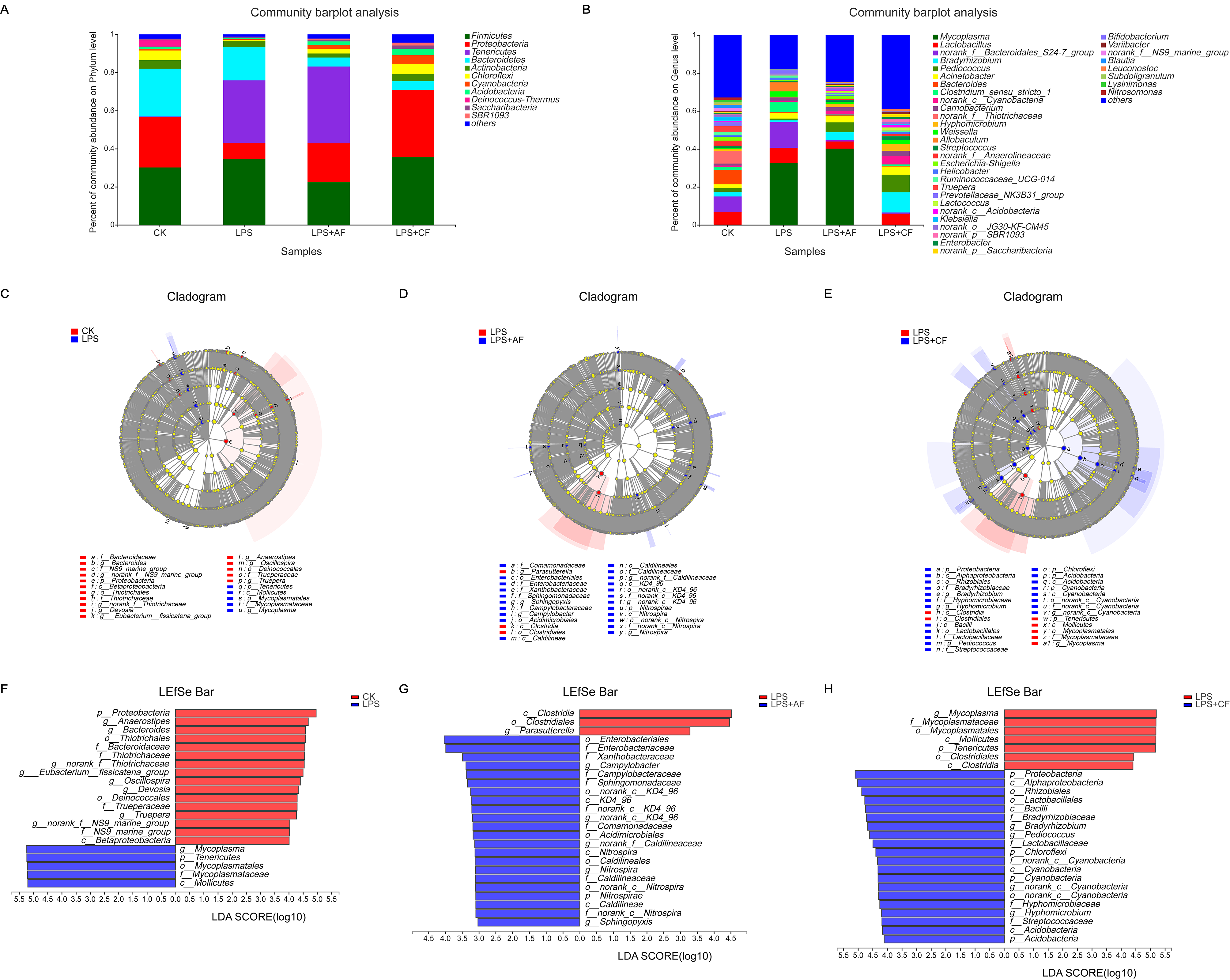

Supplement: FIG S2 [file msystems.01374-20_sf002.tif]

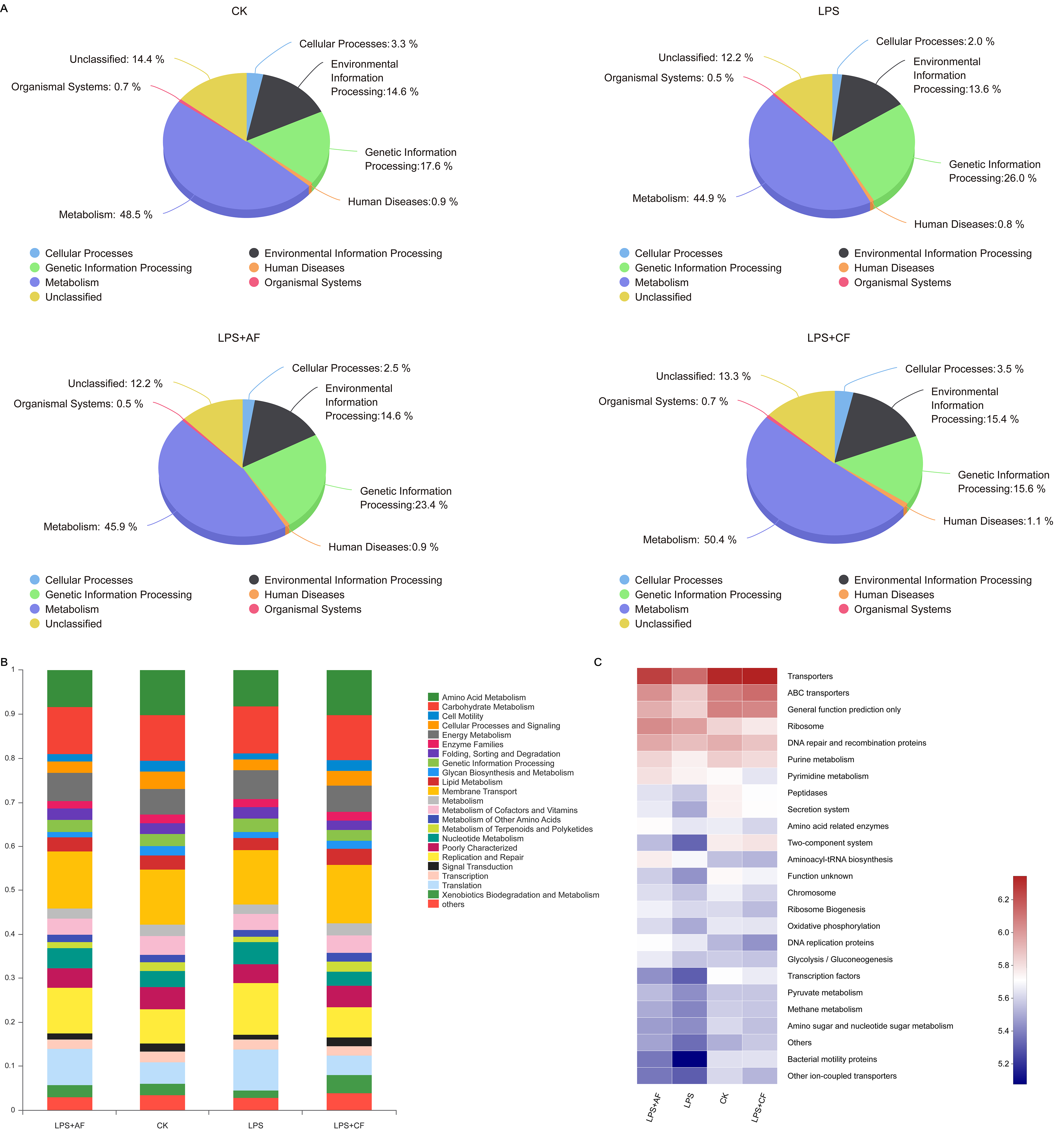

Supplement: FIG S3 [file msystems.01374-20_sf003.tif]
