# Supplementary material for: Dietary Fiber Ameliorates Lipopolysaccharide-Induced Intestinal Barrier Function Damage in Piglets by Modulation of Intestinal Microbiome
Source: mSystems. 2021 Apr 6;6(2):e01374-20. doi: 10.1128/mSystems.01374-20 (PMC8547013; doi:10.1128/mSystems.01374-20)
Supplement: TABLE S1 [file msystems.01374-20_st001.docx]

**TABLE S1** Feed composition and nutrients offered to piglets during experiment

| Items | CK | LPS | LPS+AF | LPS+CF |
| --- | --- | --- | --- | --- |
| Ingredients | | | | |
| Corn (%) | 59.37 | 59.37 | 54.93 | 56.23 |
| Soybean-puffed (%) | 11.20 | 11.20 | 11.19 | 11.08 |
| Fermented soybean meal (%) | 7.17 | 7.17 | 7.00 | 7.85 |
| Soybean meal (%) | 7.60 | 7.60 | 7.00 | 7.18 |
| Alfalfa meal (%) | - | - | 5.00 | - |
| Commodity fiber (%) | - | - | - | 2.00 |
| Fish meal (%) | 4.00 | 4.00 | 4.02 | 4.35 |
| Dried whey (%) | 5.50 | 5.50 | 4.20 | 5.00 |
| Soybean oil (%) | 1.40 | 1.40 | 3.12 | 2.62 |
| Salt (%) | 0.33 | 0.33 | 0.33 | 0.34 |
| Limestone (%) | 0.58 | 0.58 | 0.40 | 0.58 |
| Calcium hydrogen phosphate (%) | 1.17 | 1.17 | 1.12 | 1.11 |
| L-Lysine (%) | 0.32 | 0.32 | 0.32 | 0.30 |
| DL-Methionine (%) | 0.14 | 0.14 | 0.15 | 0.14 |
| Zinc oxide (%) | 0.22 | 0.22 | 0.22 | 0.22 |
| *Premix (%) | 1.00 | 1.00 | 1.00 | 1.00 |
| Total | 100 | 100 | 100 | 100 |
| Nutrient levels | | | | |
| Digestion energy (MJ·kg^-1^) | 14.65 | 14.65 | 14.65 | 14.65 |
| Crude protein (%) | 19.00 | 19.00 | 19.00 | 19.00 |
| Crude fiber (%) | 2.59 | 2.59 | 3.73 | 3.72 |
| Lysine (%) | 1.39 | 1.39 | 1.38 | 1.38 |
| Methionine+Cystine (%) | 0.76 | 0.76 | 0.76 | 0.76 |
| Calcium (%) | 0.75 | 0.75 | 0.75 | 0.75 |
| Available phosphorous (%) | 0.40 | 0.40 | 0.40 | 0.40 |

*the premix provided the following per kg of diets: Vitamin A, 5500 IU; Vitamin D3, 500 IU; Vitamin E, 66.1 IU; Vitamin B12, 28.2 μg; Riboflavin, 5.1 mg; Pantothenic acid, 12.6 mg; Nicotinic acid, 29.8 mg; Choline, 540 mg; Mn, 40 mg; Zn, 120 mg; Fe, 130 mg; Cu, 150 mg; Co, 1 mg; Se, 0.25 mg; I, 4.5 mg.
