# Supplementary material for: Dietary Fiber Ameliorates Lipopolysaccharide-Induced Intestinal Barrier Function Damage in Piglets by Modulation of Intestinal Microbiome
Source: mSystems. 2021 Apr 6;6(2):e01374-20. doi: 10.1128/mSystems.01374-20 (PMC8547013; doi:10.1128/mSystems.01374-20)
Supplement: TABLE S2 [file msystems.01374-20_st002.docx]

**TABLE S2** The primer sequence of the studied genes

| Gene | Forward primer (5′------ 3′) | Reverse primer (5′------ 3′) |
| --- | --- | --- |
| GAPDH | CGTCCCTGAGACACGATGGT | GCCTTGACTGTGCCGTGGAAT |
| Claudin-1 | TTCTGGGAGGTGCCCTACTT | TGGATAGGGCCTTGGTGTTG |
| Occludin | CCATGGCCTACTACTCGTCCAA | CGGCACCGGTGTTGATTTAT |
| ZO-1 | GACTTAAAGCTGCCTCAACAGA | GGTTTGTTTCAGGCGAAAGG |
| TLR2 | TCGAAAAGAGCCAGAAAACCAT | CTTGCACCACTCGCTCTTCA |
| TLR4 | TCAGTTCTCACCTTCCTCCTG | GTTCATTCCTCACCCAGTCTTC |
| NF-kB | AGTACCCTGAGGCTATAACTCGC | TCCGCAATGGAGGAGAAGTC |
| IL-1β | TCTGCCCTGTACCCCAACTG | CCAGGAAGACGGGCTTTTG |
| IL-6 | GATGCTTCCAATCTGGGTTCA | CACAAGACCGGTGGTGATTCT |
| TNF-α | TCCAATGGCAGAGTGGGTATG | AGCTGGTTGTCTTTCAGCTTCAC |
